# Rational design of a hydrolysis-resistant mycobacterial phosphoglycolipid antigen presented by CD1c to T cells

**DOI:** 10.1016/j.jbc.2021.101197

**Published:** 2021-09-15

**Authors:** Josephine F. Reijneveld, Laura Marino, Thinh-Phat Cao, Tan-Yun Cheng, Dennis Dam, Adam Shahine, Martin D. Witte, Dmitri V. Filippov, Sara Suliman, Gijsbert A. van der Marel, D. Branch Moody, Adriaan J. Minnaard, Jamie Rossjohn, Jeroen D.C. Codée, Ildiko Van Rhijn

**Affiliations:** 1 Division of Rheumatology, Inflammation, and Immunity, Brigham and Women's Hospital and Harvard Medical School, Boston, Massachusetts, USA; 2 Department of Infectious Diseases and Immunology, Faculty of Veterinary Medicine, Utrecht University, Utrecht, the Netherlands; 3 Stratingh Institute for Chemistry, University of Groningen, Groningen, the Netherlands; 4 Department of Bio-organic Synthesis, Faculty of Science, Leiden Institute of Chemistry, Leiden University, Leiden, the Netherlands; 5 Infection and Immunity Program and Department of Biochemistry and Molecular Biology, Biomedicine Discovery Institute, Monash University, Clayton, Victoria, Australia; 6 Australian Research Council Centre of Excellence in Advanced Molecular Imaging, Monash University, Clayton, Victoria, Australia; 7 Institute of Infection and Immunity, Cardiff University, School of Medicine, Cardiff, United Kingdom

**Keywords:** T-cell receptor (TCR), antigen presentation, glycolipid, lipid synthesis, protein crystallization, CD1c, MHC, major histocompatibility complex, MPM, mannosyl-β1-phosphomycoketide, PM, phosphomycoketide, Pks12, polyketide synthase 12, MDDC, monocyte-derived dendritic cell, TCR, T cell receptor, HD, healthy donor, GMM, glucose monomycolate

## Abstract

Whereas proteolytic cleavage is crucial for peptide presentation by classical major histocompatibility complex (MHC) proteins to T cells, glycolipids presented by CD1 molecules are typically presented in an unmodified form. However, the mycobacterial lipid antigen mannosyl-β1-phosphomycoketide (MPM) may be processed through hydrolysis in antigen presenting cells, forming mannose and phosphomycoketide (PM). To further test the hypothesis that some lipid antigens are processed, and to generate antigens that lead to defined epitopes for future tuberculosis vaccines or diagnostic tests, we aimed to create hydrolysis-resistant MPM variants that retain their antigenicity. Here, we designed and tested three different, versatile synthetic strategies to chemically stabilize MPM analogs. Crystallographic studies of CD1c complexes with these three new MPM analogs showed anchoring of the lipid tail and phosphate group that is highly comparable to nature-identical MPM, with considerable conformational flexibility for the mannose head group. MPM-3, a difluoromethylene-modified version of MPM that is resistant to hydrolysis, showed altered recognition by cells, but not by CD1c proteins, supporting the cellular antigen processing hypothesis. Furthermore, the synthetic analogs elicited T cell responses that were cross-reactive with nature-identical MPM, fulfilling important requirements for future clinical use.

The ability of T cells to respond to peptides presented by major histocompatibility complex (MHC) proteins is more widely known than their ability to respond to nonclassical MHC class I-like CD1 proteins. Whereas mice only express CD1d, humans express a functionally diverse family of CD1 antigen presenting molecules, namely CD1a, CD1b, CD1c, and CD1d (1). Unlike MHC genes, CD1 genes are essentially monomorphic, so all individuals express the same CD1 genes. The nonpolymorphic nature of CD1 proteins likely enables them to present identical antigens in all individuals, which makes ligands of CD1 proteins attractive candidates for subunit vaccine and diagnostics development. Several lipid antigens that are presented by CD1 and recognized by T cells are found in the cell wall of *Mycobacterium tuberculosis*. Here we focus on mycoketides, a class of mycobacterial lipids presented by CD1c. CD1c proteins are constitutively expressed on the surface of marginal zone and mantle zone B cells in spleen and lymph nodes, activated B cells in blood (2, 3, 4), and CD1c is a defining marker of subsets of dendritic cells (5, 6). CD1c expression can also be induced on monocytes after exposure to activating signals or the cytokines IL-4 and GM-CSF or IL-1β (7, 8).

Binding of mycoketides in the antigen-binding cleft of CD1c requires the unique mycobacteria-specific lipid branching patterns that are introduced by the enzyme polyketide synthase 12 (Pks12) (9). The Pks12 enzyme and other proteins in the biosynthetic pathway are present in mycobacterial pathogens and absent in mammalian species. The C30–34 alkyl chains in mycoketides have methyl branches every fourth carbon, starting at C4 (9, 10, 11). Two structurally related mycoketides are antigenic to T cells, mannosyl-β1-phosphomycoketide (MPM), and phosphomycoketide (PM) (10, 11, 12, 13, 14). Although present in small quantities in the *M. tuberculosis* cell wall, MPM is a potent inducer of T cell activation (10). Certain T cell responses to MPM require both a phosphate and a β-linked mannose unit (12), which protrude from the ligand binding groove of CD1c (14). Thus MPM-specific T cells, like many other glycolipid-specific T cells, are highly specific for small molecular details of the carbohydrate headgroup of their cognate antigen.

Interestingly, the human T cell line DN6 (15) responds to MPM and its deglycosylated form PM in assays using live antigen presenting cells such as monocyte-derived dendritic cells (MDDCs). However, bypassing the cellular component of antigen presentation, using plate-bound CD1c, it was shown that the T cell receptor (TCR) was specific for PM and not MPM (12). These studies suggested that MPM underwent “lipid antigen processing” to form PM by the cellular CD1c antigen presentation machinery, raising the questions whether lipid antigen processing by cells occurs generally and whether lipid antigens provided in vaccines or *in vitro* diagnostic tests might be recognized in another form. Newly synthesized CD1 molecules follow the secretory pathway to the cell membrane, after which they undergo internalization to endocytic compartments and recycle to the surface (16), where they present the lipids to T cells (17, 18). The endocytic pathway provides low pH and contains proteases, glycosidases, and lipases, which can process antigen or destroy antigenic epitopes. In particular, the phosphate ester that is the proposed site of cleavage of MPM to PM (and also links many other phospholipid head groups to their lipid anchors) is sensitive to acid or enzymatic cleavage by hydrolysis, an effect that can in theory be blocked through chemical engineering to remove the ester. However, re-engineering of the head group might also affect T cell recognition.

In the current study, we tested the MPM processing hypothesis through design and synthesis of hydrolysis-resistant analogs and T cell assays that do or do not allow cellular cleavage. We generated and identified a hydrolysis-resistant, antigenic MPM analog and provide a detailed study of epitope recognition by solving CD1c crystal structures bound to three different synthetic analogs. More practically, induction of T cell responses *in vitro* invites a new phase of research in which CD1c-presented lipid antigens are developed as immunogens. The relevance of processing of intact molecules into recognized epitopes is poorly understood in the CD1 system. Although the continuous production of MPM during a live infection with *M. tuberculosis* leads to T cell responses against MPM *in vivo* in humans (10), the processing of MPM to PM by antigen presenting cells such as dendritic cells might reduce the availability of MPM *in vivo* when applied as a subunit vaccine. Thus, the current study also provides a defined and stable epitope that is nature-equivalent for use in future vaccines and diagnostic tests.

## Results

### Design and synthesis of stabilized MPM analogs

**Figure 1:**
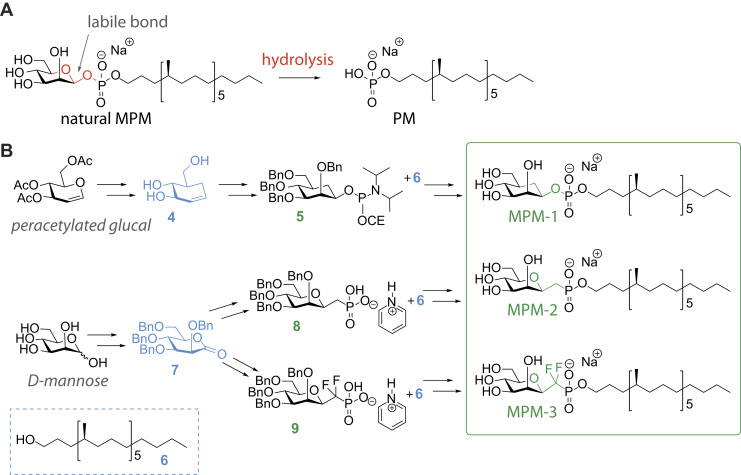
**Synthesis of stabilized MPM analogs.***A*, natural MPM contains a β-glycosidic bond, which may be subject to hydrolysis in the presence of enzymes or strong acids, leading to formation of PM. *B*, synthetic approach to generation of carba-mannose (MPM-1) and two C-mannosides (MPM-2 and MPM-3), which can be considered glycomimetics of MPM. Structural modifications, responsible for improved stability toward chemical and enzymatic hydrolysis, are highlighted in *green*. Key intermediates are highlighted in *blue*.

Enzymatic or nonenzymatic acid hydrolysis of the antigen MPM removes the mannose and gives rise to a different molecule that is also antigenic, PM (Fig. 1*A*) (12). Here we set out to generate MPM analogs with improved stability by creating hydrolysis-resistant bonds between the sugar and phospholipid moiety, while still preserving recognition. Previously, through stereoselective total synthesis of a β-D-mannosyl phosphomycoketide, we have shown the importance of the stereochemistry of the lipid moiety in phosphomycoketides for T cell response (19). The synthetic mycoketide lipid moiety was used here to generate three new MPM analogs with predicted chemical stability. These synthetic analogs comprise a carba-mannose, where the ring oxygen is replaced by a methylene group (MPM-1), and two C-mannosides, where the anomeric oxygen is replaced by a methylene group or a difluoro methylene moiety (MPM-2 and MPM-3, respectively) (Fig. 1*B*). Altering the structure of the mannose moiety of MPM to generate the more stable glycomimetic might impact CD1c-binding and T-cell recognition, but it is difficult to predict how the different modifications will affect the interaction with both binding partners. Replacing the acetal linkage by an ether functionality will result in conformational changes of the key exocyclic phosphate substituent as the exo-anomeric effect that determines the orientation of the exocyclic substituent is missing in all three stabilized analogs. The overall dipole of the molecules will also be different as well as hydrogen bonding and accepting properties. Finally, the pKa of the phosphate in the analogs will differ from the parent compound. These effects should be minimized in C-mannoside MPM-3, since the difluoro methylene has most similar electronic properties compared with those of the anomeric oxygen, present in the naturally occurring MPM (20).

The synthetic strategies for the MPM analogs were designed to avoid the use of and formation of PM during reaction or purification steps. Thus, for the generation of MPM-1, a phosphoramidite chemistry approach was chosen to couple lipid **6** to β-carba-mannopyranoside **5**, which in turn was obtained from key pseudo-glucal intermediate **4**. The two C-glycosides MPM-2 and MPM-3 were synthesized starting from d-mannose *via* lactone **7**, which was converted to phosphonates **8** and **9** *via* exo-glycal and lactol intermediates, respectively. Inspired by previous work on glucose (21), we implemented a hydrophosphonylation reaction to synthesize the desired β-C-mannoside **8**, of which we demonstrated the intended stereochemical outcome (Fig. S1). The coupling reactions between phosphonate **8** or **9** and lipid **6** yielded the desired analogs MPM-2 and MPM-3. Our method for coupling of difluoro-C-mannoside **9** to the lipid required the use of an excess of lipid **6** to prevent pyrophosphate formation. All products were validated by NMR and MS (Data S1).

The purification steps for sensitive intermediates and final products were performed avoiding acidic conditions, and compounds were stored dry at –20 °C. Even upon long-term storage, chemical hydrolysis of the two C-mannopyranoside analogs (MPM-2 and MPM-3) can never lead to formation of PM, as lipid **6** would be the hydrolysis product. The expected stability of all three analogs toward mannosidase enzymes is based on the mechanism of action by which these enzymes remove mannosides from their substrates. Glycosidase-induced hydrolysis is effected through reactions proceeding with significant oxocarbenium ion character (22), the formation of which is not possible once the carbohydrate acetal is replaced by the ether functionality in our carba- and C-glycosides. It is however not possible to exclude hydrolysis of the anomeric phosphodiester induced by phosphatases in the setting of *in vitro* antigen-presentation experiments with dendritic cells. This would lead to formation of PM from carba-analog MPM-1 or lipid **6** for C-mannopyranoside analogs MPM-2 and MPM-3. Thus, depending on the active pathways of degradation in antigen presenting cells, one or more MPM analogs may be resistant to degradation.

### MPM-3 withstands processing to PM

**Figure 2:**
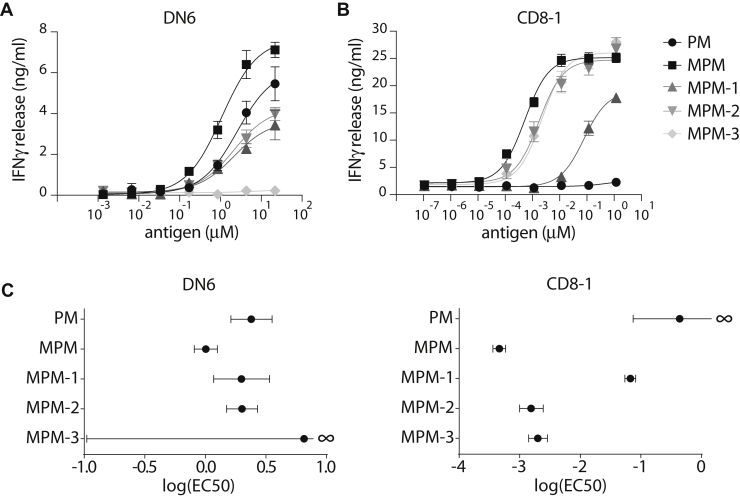
**MPM-3 withstands hydrolysis and cross-reacts with natural MPM.***A*, IFNγ production by PM-specific DN6 T cells cocultured with MDDCs in the presence of natural MPM and MPM analogs. *B*, IFNγ production by MPM-specific CD8-1 T cells cocultured with MDDCs in the presence of natural MPM or MPM analogs. Data are mean ± SD of triplicate measurements representative of three experiments. Data were fitted to a three parameter dose response curve. *C*, summary statistics with half maximal effective concentrations (EC50) and their 95% confidence interval (CI).

To study the occurrence of antigen modification by antigen-presenting cells, we made use of two established human T cell lines, DN6 and CD8-1 (15, 23). The DN6 TCR is specific for PM and responds to natural or nature-identical synthetic MPM after cellular processing to PM. The β-mannose unit is neither required nor permissive for activation of the DN6 cell line (12). MPM-3 did not induce an interferon-γ (IFNγ) response, as expected based on its inability to give rise to PM due to the stabilized bond between sugar and lipid. However, MPM-1 and MPM-2 were both able to activate DN6 T cells (Fig. 2*A*), which could be caused by cross-reactive recognition of the intact compounds by the TCR, or by the presence of PM, which could be generated by hydrolysis during storage or in antigen-presenting cells. In an initial experiment, using high-performance liquid chromatography–mass spectrometry (HPLC–MS), we found no detectable PM in our MPM stocks (Fig. S2*A*). To test the possibility of PM generation by antigen-presenting cells, we treated MDDCs with MPM or its analogs, followed by a total lipid extraction from the cells and analysis by HPLC-MS. We detected a trace amount of PM in our MPM and MPM-1 stocks and detected higher amount of PM in the lipid extracts of MPM or MPM-1-treated cells. MPM-2 and MPM-3 did not give rise to any detectable PM in either the stock solutions or the cell extracts (Fig. S2*B*). Thus, the formation of PM from MPM or MPM-1 during storage or in antigen presenting cells can account for the activation of the DN6 cells. For MPM-2, we could not detect formation of PM, and the likely mechanism of recognition by DN6 is cross-reactive recognition of the intact compound. MPM-3 does not stimulate a cross-reactive response by the PM-specific TCR DN6, nor does it give rise to PM, demonstrating successful blockade of its processing.

### Antigenic equivalence of natural MPM, MPM-2, and MPM-3

The CD8-1 TCR is highly specific for the β-linked mannose headgroup of MPM (10, 12). For the stabilized MPM analogs to be useful in future applications such as diagnostics or vaccines, they should cross-react with natural MPM, despite the chemical changes in the headgroups. Therefore, we tested response of the CD8-1 T cell line to the stabilized MPM analogs. Both MPM-2 and MPM-3 elicited a response similar to nature-identical MPM, indicating that the chemical modification did not influence recognition by the TCR (Fig. 2*B*). Recognition of MPM-1, however, was reduced, as seen by a reduction in IFNγ expression by CD8-1 T cells and significantly increased EC50 (Fig. 2*C*). Overall, these results show that the analog MPM-3 is able to withstand processing to PM, while remaining antigenic for the CD8-1 TCR.

### Molecular detail of MPM analog presentation by CD1c

**Figure 3:**
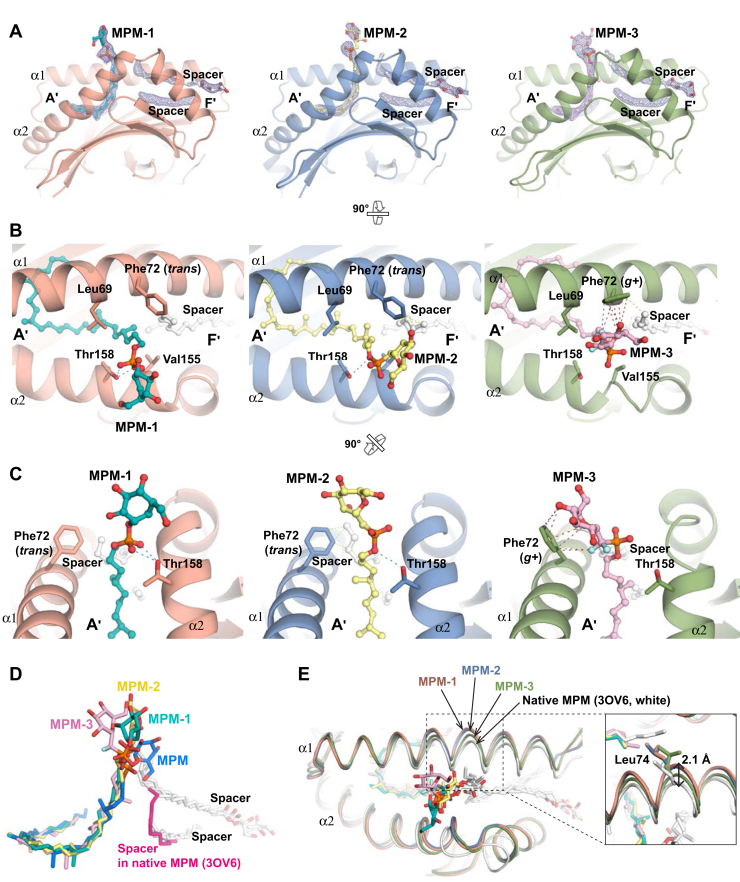
**Binary structures of CD1c with MPM analogs.***A*, front view of CD1c with MPM analogs. Electron densities of ligands are contoured at 0.75 σ. Cartoon representation is colored as *salmon*, *blue*, and *green* for structure with MPM-1, MPM-2, and MPM-3, respectively. MPM analog models are depicted as sticks with balls colored with *aquamarine*, *yellow*, and *pink* for MPM-1, MPM-2, and MPM-3, respectively. Spacer lipids are in *white*. *B*, top views of CD1c with MPM analogs show the positions of MPM analogs' headgroups. Color codes are same as in (*A*). *C*, side view of CD1c with MPM analogs from A′ pocket is showing the orientation of MPM analogs' headgroup. Color codes are same as in (*A*) and (*B*). *D*, superposition of MPM analogs with native MPM (3OV6). The three MPM analogs are depicted as sticks with the same color codes in (*A*), (*B*), and (*C*). The native MPM is depicted as *marine-blue stick*, while its spacer lipid is colored in *hot-pink*. *E*, superposition of CD1c binary structures together with native MPM structure (*white*, 3OV6) is showing the movement of helices upon ligand binding.

To determine the mechanism of epitope display, we determined binary crystal structures of CD1c-MPM-1, -MPM-2, and MPM-3 at 1.7, 2.0, and 2.1 Å, respectively (Table S1). In addition to electron density corresponding to the MPM analogs (Fig. S3), electron density corresponding to two putative spacer lipids was observed in the F′ pocket (Fig. 3*A*), which were analogous to those seen in the published CD1c-endogenous lipid-3C8 TCR ternary structure and binary CD1c structures (24, 25). Accordingly, we modeled this “spacer” density as monoacyl glycerol, as this single tail lipid has been previously observed in the CD1c binding pocket (Fig. 3*A*) (25). The density in the A′ pocket unambiguously indicated the mycoketide tail of the MPM analogs, with clear electron density present for each of the branched methyl groups positioned and conserved in all three structures (Fig. 3, *A* and *B*).

In contrast, the conformational flexibility of the headgroups led to relatively poorly resolved electron density, presumably due to its protrusion out of the binding cleft, consistent with the published native MPM structure (3OV6) (Fig. 3*C*). Aligning with the native MPM structure (3OV6) (14) revealed that the position of the phosphate group of the MPM analogs was conserved, although being slightly reoriented (Fig. 3*D*). The phosphate group was sandwiched between a hydrophobic cluster including Leu69 and Phe72 of the α1 helix and Val155 and Thr158 of the α2 helix (Fig. 3, *B* and *C*). The phosphate was unable to establish stabilizing interactions with CD1c, except for hydrogen bond formation with the Thr158 Oγ atom. This was observed in the MPM-1 and MPM-2 structures (Fig. 3, *B* and *C*), which accounted for its reorientation within this region.

Of the three MPM-analogs, MPM-3's headgroup appeared to be best stabilized, through an interaction with Phe72. To accomplish this stabilization, Phe72 adopted a *g*+ conformation at a torsion angle of 89.6°, compared with *trans* in the other two analogs with angles of 171.3° and 181.0° for MPM-1 and MPM-2, respectively. This suggests that Phe72 played a role in the orientation and presentation of these ligands to TCRs.

We also observed a considerable movement of the CD1c α1 helix in the CD1c-MPM-1 and CD1c-MPM-2 structures compared with the CD1c-MPM-3 and native CD1c-MPM structures, with up to 2.1 Å difference at Leu74 Cα (Fig. 3*E*), which appears to be ligand-induced. These high-resolution binary structures provide evidence that CD1c is subject to conformational alterations upon ligand binding that may impact antigen presentation to T cells. Of the CD1c-MPM analogue binary structures, the structure of CD1c-MPM-3 most closely resembled that of CD1c-native MPM.

### Detection of MPM-specific T cells after *in vitro* immunization with MPM-3

**Figure 4:**
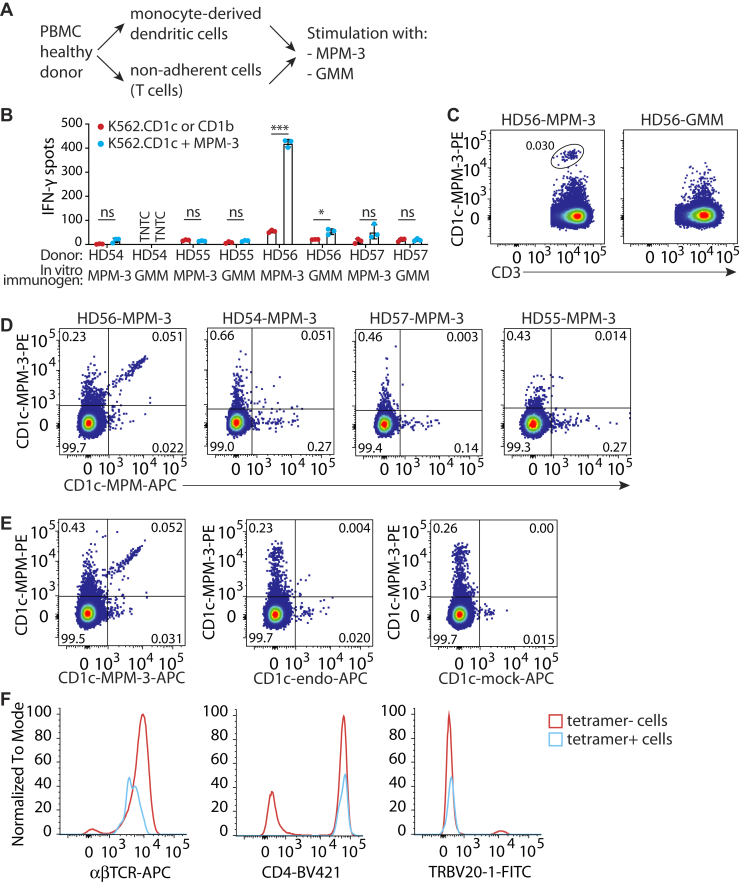
**Cross-recognition of MPM and MPM-3 by human T cells after *in vitro* immunization with MPM-3.***A*, schematic representation of stimulation of T cells from healthy donors with MDDCs incubated with either MPM-3 or negative control (GMM) lipids. *B*, IFN-γ ELISPOT screen for recognition of MPM-3 of healthy donor-derived (HD) cell lines stimulated with K562.CD1c cells in the presence of MPM-3 or GMM. Error bars represent the SEM of triplicate wells of one experiment. For each cell line, an unpaired, two-tailed *t* test was used to determine statistical significance of MPM-3 recognition. ∗*p* < 0.05, ∗∗*p* < 0.01, ∗∗∗*p* < 0.001. *C*, flow cytometry dot plots of CD1c-MPM-3 tetramer staining of lines HD56-MPM-3 and HD56-GMM. *D*, flow cytometry dot plots of double tetramer staining on line HD56-MPM-3, HD54-MPM-3, HD57-MPM-3, and HD55-MPM-3. *E*, CD1c-MPM-PE and CD1c-MPM-3-APC tetramers were used to stain HD56-MPM cells. CD1c-MPM-3 tetramers are shown plotted against CD1c-endo or CD1c-mock tetramers. *F*, flow cytometry analysis of expression of αβTCR, CD4, and TRBV20-1 by tetramer-positive and -negative cells in line HD56-MPM-3. TNTC, too numerous to count.

Because of its desirable chemical characteristics, we next tested whether we could expand MPM-specific T cells by stimulating peripheral blood mononuclear cells *ex vivo* with the analog MPM-3, a process that is called *in vitro* immunization. For four healthy donors (HD) that were most likely never exposed *to M. tuberculosis*, plastic nonadherent lymphocytes were stimulated with autologous MDDCs that were incubated with either MPM-3 or the unrelated mycobacterial lipid glucose monomycolate (GMM) as a negative control (Fig. 4*A*). After two rounds of antigen stimulation, we performed an initial screen for MPM-3 recognition by ELISPOT. We observed a strong MPM-3-dependent response in a T cell line from healthy donor 56 immunized *in vitro* with MPM-3 (HD56-MPM-3) (Fig. 4*B*). This initial positive result prompted us to develop CD1c-MPM tetramers, using the HD56-MPM-3 cell line as a positive control for staining. Although CD1c tetramers loaded with PM (12), acylated cholesteryl β-D-galactoside (24), or endogenous mammalian lipids (25) have been developed previously, CD1c-MPM tetramers have not been previously reported. Using a CD1c loading protocol based on previously published PM loading, we observed a CD1c-MPM-3 tetramer positive population in the T cell line HD56-MPM-3 that was not present in the cell line derived from the same donor by stimulation with GMM (HD56-GMM, Fig. 4*C*). The need for *in vitro* immunization with MPM-3 suggests that low frequency T cells, most likely naive T cells, were expanded and detected. Thus, we were able to detect MPM-3 tetramer positive cells in the HD56-MPM-3 cell line by ELISPOT and tetramer staining after *in vitro* immunization.

Next, we tested whether the T cells that recognize MPM-3 cross-react with natural MPM by staining cells with both CD1c-MPM-3 and CD1c-MPM tetramers. The tetramer positive population in the HD56-MPM-3 cell line recognized both natural MPM and MPM-3. In the cell lines from the other three donors, we could not detect CD1c-natural MPM- or CD1c-MPM-3-tetramer positive cells (Fig. 4*D*). Of note, both tetramers stained double positive cells in the HD56-MPM-3 cell line brightly. The mean fluorescence intensities were equivalent, even when fluorophores were swapped on the tetramers, suggesting equivalent TCR binding to CD1c in complex with natural MPM or MPM-3.

Other T cells have been reported that recognize CD1c complexes carrying endogenous phospholipids (CD1c-endo) from the mammalian CD1 protein expression system. Such T cells recognize CD1c-phospholipid or bind the CD1c protein itself (25, 26). Therefore, we also stained the T cells simultaneously with an untreated CD1c tetramer (CD1c-endo) and a mock-treated CD1c tetramer (CD1c-mock), which showed that most CD1c-MPM-3 tetramer-binding cells fail to bind CD1c-endo (Fig. 4*E*). Therefore, unlike recently discovered T cells that directly recognize CD1a (27) or CD1c (25), the MPM-tetramer positive cells we detected are specific toward the antigen loaded in CD1c and not the protein itself.

To gain more insight in the CD1c-MPM-binding cells, we analyzed their TCR type and CD4 or CD8 expression. The MPM-tetramer positive cells stained positive for αβTCR and CD4 (Fig. 4*F*). Some antigen-specific T cells show a bias toward TRAV and TRBV usage. Therefore, we stained the cells for TRBV20-1, which is the Vβ chain used by the CD8-1 T cell line that is specific for MPM. The tetramer-binding cells did not express TRBV20-1 (Fig. 4*F*). Thus, *in vitro* immunization with MPM-3 generates αβ T cells that cross-reactively recognize MPM, and these cells express a TCR that is different from the established CD8-1 clone.

### MPM is less active than MPM-3 in a cellular antigen presentation assay

**Figure 5:**
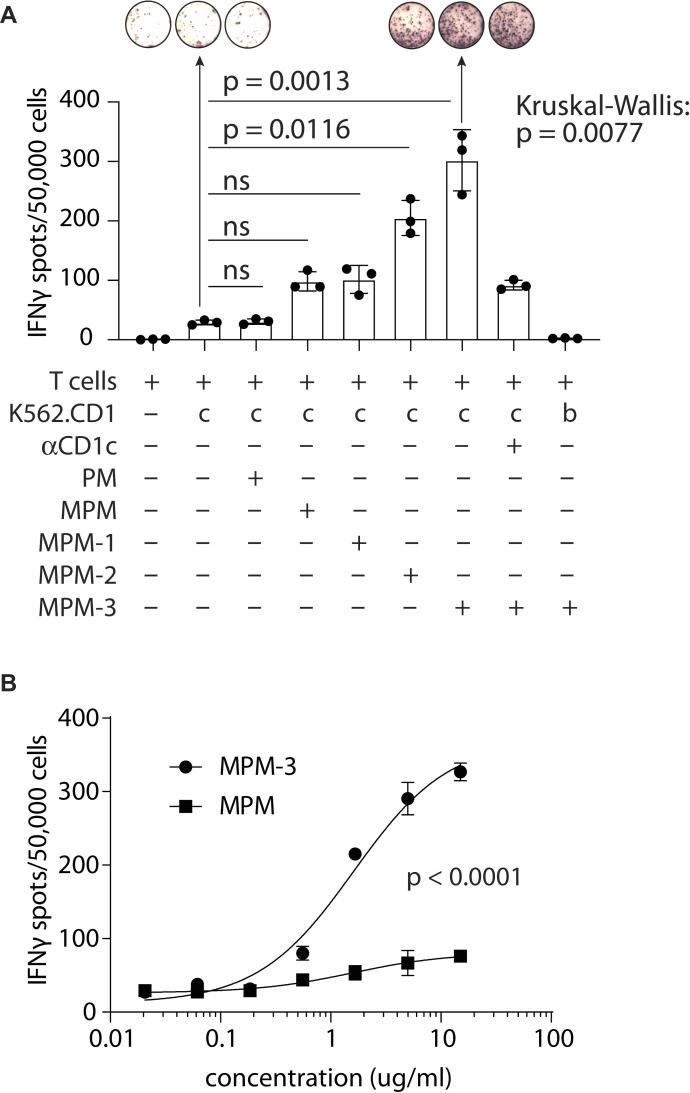
**HD56-MPM-3 T cells produce IFNγ after stimulation with MPM-3 and nature identical MPM.***A*, IFN-γ ELISPOT of the HD56 cell line stimulated with K562.CD1c or K562.CD1b cells in the presence of the indicated lipid antigens. Error bars represent the SEM of triplicate wells. One representative experiment of three is shown. The significance of the added lipids on IFN-y spots was analyzed by one-way ANOVA (Kruskal–Wallis), followed by a Dunn's post-test comparing antigen-stimulated conditions to the no antigen control consisting of T cells and K562.CD1c. *B*, dose–response curves of HD56 stimulated with K562.CD1c and lipid antigens. Data were fitted to a three parameter dose–response curve. The null hypothesis that both datasets could be described by the same curve was tested by an extra-sum-of-squares F test.

Although tetramer staining of HD56-MPM-3 suggested equivalent binding of the antigen-specific T cells for MPM and MPM-3, we wanted to compare responses against natural MPM, its breakdown product PM, and the three MPM analogs in a cellular antigen presentation assay. HD56-MPM-3 T cells stimulated with the line K562 transfected with CD1c (K562.CD1c) and MPM-3 secreted IFNγ. Nature-identical MPM induced IFNγ release, but to a lesser extent (Fig. 5). MPM-1 and MPM-2 also induced a response, with MPM-1 eliciting the lowest response, similar to that of natural MPM. MPM-2 showed an intermediate response between MPM-1 and MPM-3. PM was not recognized above background levels, and K562.CD1b cells treated with MPM-3 did not stimulate, making clear that the mannose unit and CD1c were required. Activation by MPM-3 and K562.CD1c was strongly inhibited by anti-CD1c (77% inhibition), indicating that activation of the T cells is mediated by CD1c.

Despite highly similar binding of CD1c-MPM and CD1c-MPM-3 tetramers (Fig. 4, *D* and *E*), we expected a lower stimulation by MPM in cellular assays because of the known hydrolysis of MPM into PM in antigen-presenting cells. An alternative explanation for the observed different performance at a fixed concentration is an inaccuracy of quantification or toxicity of lipids at 15 μg/ml. However, a comparison of dose–response curves of HD56-MPM-3 cells stimulated with MPM or MPM-3 (Fig. 5*B*) demonstrates that MPM is less stimulatory at every concentration tested and thus less active than MPM-3 in a cellular antigen presentation assay. This finding is consistent with the conclusion that hydrolysis of MPM to PM reduces the amount of available antigen, while MPM-3 is not sensitive to hydrolysis. This favored interpretation is separately supported by plate-bound antigen presentation assays that distinguish responses to PM and MPM (12), the known susceptibility of this type of glycosyl phosphate diesters to hydrolysis (28), the demonstrated hydrolysis of MPM, and the overcoming of antigen loss by substituting the link between sugar and phosphate with alternatives that are not subject to hydrolysis.

## Discussion

Here we applied rational antigen design to create a compound that stimulates human T cell responses against the *M. tuberculosis*-derived glycolipid MPM. Two factors were considered in the design: stability of the antigen and its ability to generate a response that cross-reacts with the natural lipid. For peptides, such studies are typically performed by systematically mutating amino acids in a peptide sequence using standard peptide synthesis technology. For lipids, the possible variations are only confined by the limitations of chemical synthesis, for which a method has to be developed for each desired type of modification. For the lipids at hand, we developed effective routes of synthesis to assemble the three types of analogs. We focused exclusively on the hydrophilic head and phosphate neck region of MPM because changes in the tails of lipid antigens have previously been shown destabilize the complex between lipid and CD1 (29) or diminish antigenicity (11). This is the first time that neck and headgroup modifications for CD1c-presented antigen have been designed, synthesized, and tested.

Among the few known processed lipid antigens (30, 31), MPM is a highly useful experimental model because T cells recognizing processed and unprocessed antigens are available (12), nature-identical and altered mycoketides are accessible by synthesis (19), and the mode of presentation is known from a prior study (14) and three new CD1c-mycoketide lipid structures reported here. Once the mannose has been removed, the lipid loses its antigenicity for a T cell line that is specific for intact MPM. We designed modifications in the neck and headgroup that we expected to confer resistance to mannosidase, acid-mediated hydrolysis, or other cellular mechanisms that attack the phosphate ester, realizing that the modifications could also destroy antigenicity. Of the three MPM analogs that we designed and synthesized, MPM-3 was completely insensitive to degradation, and it was fully cross-reactive with natural MPM by several criteria, while the other two analogs had intermediate characteristics. Protein crystallography demonstrated that the phosphate group of MPM-3 was highly stabilized in a configuration comparable to natural MPM, which was supported by the difluoro methylene group. The difluoro methylene itself was turned away from the CD1c surface, which may be the reason that it does not interfere with TCR docking. In addition, the difluoro methylene causes no significant change in pKa of the molecule so that it carries a negative charge under physiologic conditions, like natural MPM.

The lipid tails and phosphate of all three MPM analogs were anchored to the CD1c protein. Despite this, the mannose headgroup of all three MPM analogs showed considerable conformational flexibility. Despite the limited overlap, the antigenicity of natural MPM and MPM-3 is highly comparable. A potential explanation for this observation is that TCR docking may stabilize the headgroups. The anchoring of phosphates to CD1c is different from how phospholipids are presented by CD1b, where phosphate groups are not stabilized by interactions with CD1b, but rather by the TCR, in the so-called “cationic cup” in the TCR (32, 33). In both CD1b and CD1c, the phospholipid headgroups are flexible in the binary CD1-lipid structure, and in the case of CD1b it was demonstrated that the headgroup was remodeled and anchored by the TCR upon TCR docking (33).

Overall, the work presented here provides detailed insights into presentation of MPM by CD1c and provides three key outcomes. First, we have developed a CD1c-MPM tetramer that can be used to detect human MPM-specific T cells in people infected with *M. tuberculosis*. It was previously demonstrated that T cell responses against MPM are stronger in infected *versus* uninfected humans (10). Tetramers will enable us to analyze TCR repertoire and immunophenotype these cells and assess their contribution to protection against tuberculosis. Second, we provide versatile synthetic strategies to obtain stabilized analogs of β-mannoses, which can now be employed to generate carba-mannose, C-mannoside, and difluro-C-mannoside for virtually any natural β-mannose with a phosphate in the anomeric position. Last, we have created an MPM analog, MPM-3, that is nature-equivalent MPM, but not susceptible to enzymatic cleavage.

MPM-3 can now be used in preliminary immunization studies in CD1c-expressing species, such as human CD1c transgenic mice or guinea pigs that naturally express several CD1c genes. The inclusion of MPM-3 in an experimental vaccine is expected to broaden the T cell response against *M. tuberculosis* as compared with immunization with PM or hydrolyzable forms of MPM only, which is desirable because broader T cell responses are generally associated with better protection (34). Further, generation of durable mannose epitopes reduces uncertainty about which epitopes are recognized *in vivo*, and we can use MPM-loaded tetramers to measure immunological function. If animal studies demonstrate immunogenicity of MPM-3 *in vivo*, they will be followed up by immunization-challenge experiments with *M. tuberculosis,* ultimately paving the way for its use in subunit vaccines.

## Experimental prodecures

### Synthesis of MPM analogs

Details of MPM analog synthesis are provided in Data S1. For C-mannosyl-1-phosphomycoketide (MPM-1), pseudo-glucal intermediate was prepared from peracetylated D-glucal using a similar approach to that published by Gao *et al.* (35). A benzylidene was installed and the olefin was then epoxidized to give an α:β mixture of epoxides (1:17). Both epoxides reacted with hydroxide to give the desired pseudo-mannoside configuration. The obtained α-mannopyranoside was converted to β-mannopyranoside *via* an oxidation/reduction procedure. After protecting groups manipulation, the carbasugar phosphoramidite was synthesized. Coupling to lipid alcohol followed by final deprotection steps resulted in the synthesis of the desired final product with a 1.4% yield over 19 steps starting from peracetylated D-glucal. For mannose-1-C-phosphonate mycoketide (MPM-2), manno-lactone intermediate was prepared from D-mannose as described by Waschke *et al.* (36) and subjected to Petasis reaction for the generation of key intermediate exoglycal. This intermediate was used in a UV-assisted radical hydrophosphonylation reaction (21) followed by deprotection of the newly synthesized phosphonate. NMR analysis, including NOESY measurements on the C-mannosyl methyl-deprotected phosphonate, was used to confirm β-stereochemistry of the product formed by radical hydrophosphonylation. The coupling of benzyl-protected C-mannosyl phosphonate with lipid alcohol was followed by hydrogenation to remove benzyl-protecting groups, resulting in the desired final product with an overall yield of 6.7% over ten steps starting from D-mannose. For mannose-1-C-difluorophosphonate mycoketide (MPM-3), manno-lactone intermediate (see (36)) was subjected to nucleophilic addition, followed by removal of the newly formed anomeric hydroxyl *via* formation of intermediate methyloxalate, which was subsequently reduced using Bu_3_SnH. As for the C-mannoside analog, β-product formation was confirmed by analysis of NOESY-NMR after phosphonate deprotection. With the free phosponate acid available, a coupling reaction in the presence of tri-iso-propylphenylsulfonyl chloride was performed. Final debenzylation yielded desired C-mannopyranoside with an overall yield of 15% over six steps starting from manno-lactone.

### Cell lines

PBMCs were obtained from deidentified leukoreduction collars provided by the Brigham and Women's Hospital Specimen Bank, as approved by the Partners Healthcare Institutional Review Board. MDDCs were obtained from human PBMC by adherence to plastic after 1 h, followed by treatment with 300 IU/ml granulocyte-macrophage colony-stimulating factor and 200 IU/ml IL-4 for 72–96 h. The nonadherent fraction containing T cells was frozen as well. For antigen stimulation, the nonadherent T cells were stimulated with autologous MDDCs (2:1) in the presence of MPM-3 or GMM (2 μg/ml). The next day IL-2 was added to the culture. After 2 weeks, cell lines were analyzed for response to lipid. Expansion of derived cell lines after two rounds of antigen stimulation was performed by culturing 0.5–1 × 10^6^ T cells in a T25 flask containing 25 × 10^6^ irradiated allogeneic PBMCs, 5 × 10^6^ irradiated Epstein–Barr virus transformed B cells, and 30 ng/ml anti-CD3 antibody (clone OKT3). The next day human IL-2 was added to the culture. After 3 weeks, the stimulation or expansion procedure was repeated as needed.

### Functional T cell assays

The CD1c-MPM reactive T cell line CD8-1 (23) and CD1c-PM reactive cell line DN6 (15) were tested for IFNγ release by enzyme-linked immunosorbent assay (ELISA). 5 × 10^4^ T cells were incubated with 5 × 10^4^ APCs in 96-well culture plates (0.2 ml/well) with titrating concentrations of synthetic lipid for 24 h (37 °C, 5% CO_2_). Corning 96-well Clear Flat Bottom Polystyrene High Bind Microplates were coated with IFNγ coating antibody (2G1, Invitrogen, 2 μg/ml) overnight and blocked with 1% BSA. Supernatant was added to plates and incubated for 1 h at room temperature (RT) followed by detection with biotinylated IFNγ antibody (B133.5, Invitrogen, 0.1 μg/ml) and streptavidin-HRP. The reaction was visualized by the addition of ABTS, and absorbance was measured at 405 nm using an ELISA plate reader. For enzyme-linked immune absorbent spot (ELISPOT) assays, cocultures of 2 × 10^4^ APCs and 5 × 10^4^ T cells were incubated with MPM or GMM (15 μg/ml) for 20 h in a Multiscreen-IP filter plate (96 wells; Millipore) coated and developed according to the manufacturer's instructions (Mabtech). For blocking of CD1c, the APCs were preincubated with anti-CD1c (F10.21A3) for 15 min at 37 °C before addition of T cells. Visual representation and statistical analyses of T cell assays were performed with Graphpad Prism 9.2.0.

### Tetramers

For tetramer assays, biotinylated WT CD1c monomers were obtained from the National Institute of Health Tetramer Core Facility. CD1c monomers were loaded with MPM at a 50-fold molar excess of lipid to protein. In a 10 mm wide glass tube CD1c monomers were incubated with natural MPM or MPM-3 in phosphate buffered saline pH 7.4 for 18–24 h at 37 °C. For CD1c-mock tetramers, the treatment was the same, but no lipid was added to the tube. For CD1c-endo tetramers, the monomers were diluted in PBS (0.2 mg/ml) and tetramerized. Monomers were tetramerized using streptavidin-APC (Molecular Probes) or streptavidin- PE (Invitrogen).

### Flow cytometry

Human T-cell lines were stained with tetramers at 2 μg/ml in PBS containing 1% BSA and 0.01% sodium azide. Cells were preincubated with unlabeled CD36 antibody (5-271; Biolegend) for 10 min at RT (37). Tetramer was added and incubated for 10 min at RT in the dark, followed by addition of cell surface antibodies for 10 min at RT. Subsequently, cells were treated with unlabeled OKT3 antibody for 5 min at RT followed by 15–20 min at 4 °C. Cells were analyzed using the BD LSRFortessa flow cytometer and FlowJo software. Antibodies that were used: CD3-BV421 (UCHT1; Biolegend), αβTCR-APC (IP26; Biolegend), CD4-BV421 (OKT4, Biolegend), TRBV20-1-FITC (MPB2D5, Beckman Coulter).

### Antigen processing assay

To determine processing of MPM analogs, we incubated 2 × 10^6^ MDDCs for 24 h in 2 ml medium supplemented with 300 IU/ml granulocyte-macrophage colony-stimulating factor, 200 IU/ml IL-4, and 20 μM lipid (PM, MPM, MPM-1, MPM-2, or MPM-3). Cells were resuspended, pelleted, washed twice with PBS, and transferred to glass tubes before extraction with chloroform/methanol 1:2 for 2 h followed by chloroform/methanol 2:1 for another 2 h. Two sequential extractions were combined and dried under nitrogen. Quarter of the lipid extracts (equivalent to 5 × 10^6^ cells) were redissolved in the starting HPLC mobile phase and subjected to mass spectrometry negative mode analysis using an Agilent 6546 Accurate-Mass Q-TOF and 1260 series HPLC system with a normal phase Inertsil Diol column (150 mm × 2.1 mm, 3 μM; GL Sciences) and a guard column (10 mm × 3 mm, 3 μM; GL Sciences), running at 0.15 ml/min as the published method (38).

### CD1c expression for crystallization

For crystallization, a CD1c chimera construct was designed based on the previous method of Scharf *et al*. (14) in which the α3 domain of CD1c was swapped with that of CD1b, and three putative glycosylated asparagine sites, Asn52, Asn57, and Asn128, were mutated to glutamine, leaving the other two putative sites, Asn20 and Asn241, intact. Two additional mutations were made to prevent steric clash (Trp242Gly) and facilitate crystallization (Lys108Gly). Optimized coding sequence of the chimeric CD1c ectodomain was fused linked to β2m with P2A self-cleaving peptide and cloned into pHLSEC vector. The N-terminal IgK leading sequence was utilized to facilitate the expression of the construct in Hek293S cells, while C-terminal 6xHis-tag was used to facilitate the purification, as previously described (25). Secreted CD1c associated with β2m was harvested and proceeded to serial purification steps including Nickel affinity, ion exchange and size-exclusion chromatography. Final purified product was buffer exchanged into 10 mM Tris and 150 mM NaCl, pH 8.0. For lipid loading, native MPM and its three analogs were dissolved in 10 mM HEPES and 150 mM NaCl, pH 7.4 supplemented with 0.5% tyloxapol. Prior to loading with target lipids, CD1c was treated with an additional 1% tyloxapol for 1 h at 37 °C. The desired lipid was then added to the protein at molar ratio 10:1 for 16 h at 37 °C. Excess lipid and tyloxapol were removed by anion exchange chromatography and lipid loading into CD1c validated by isoelectric focusing electrophoresis (IEF).

### Crystallization and structure determination

Chimeric CD1c loaded with lipid was treated with thrombin to remove its C-terminal 6xHis-tag and Endo Hf to deglycosylate the Asn20 and Asn241. The protein samples were concentrated to 5 μg/μl for hanging drop crystallization in 0.1 M CHES pH 9.4, 1 M trisodium citrate, and 25 mM triglycine at 20 °C. Optimal crystals were cryoprotected in 50% sodium malonate, then flash-frozen in liquid nitrogen before data collection was conducted at the MX2 beamline at the Australian Synchrotron, part of ANTSO, and made use of the Australian Cancer Research Foundation (ACRF) detector (39). Data processing, space group determination, merging, and scaling were carried out using XDS (40), Pointless, Aimless, and C-truncate (41, 42). Molecular replacement was proceeded using Phaser (43) (Phenix suite) with CD1c-MPM structure (PDB ID: 3OV6) (14) as reference model. MPM analog models were generated using JLigand (44) and phenix.elbow (45). After manual building in Coot (46), structures were proceeded to several rounds of refinement using phenix.refine (47) and validated using MolProbity (48).

## Data availibility

The datasets generated and analyzed during the current study were deposited to the Protein Data Bank under codes 7MX4, 7MXF, and 7MXH. Information and requests for resources should be directed to and will be fulfilled by the lead contact, I. V. R. (i.vanrhijn@uu.nl).

## Supporting information

This article contains supporting information (36, 49, 50, 51).

## Conflict of interest

The authors declare that they have no conflicts of interest with the contents of this article.
