# Supplementary material for: Rational design of a hydrolysis-resistant mycobacterial phosphoglycolipid antigen presented by CD1c to T cells
Source: J Biol Chem. 2021 Sep 15;297(4):101197. doi: 10.1016/j.jbc.2021.101197 (PMC8511953; doi:10.1016/j.jbc.2021.101197)
Supplement: Supporting information [file mmc1.pdf]

Rational design of a hydrolysis-resistant phosphoglycolipid antigen presented by CD1c to human T cells

Josephine F. Reijneveld, Laura Marino, Thinh-Phat Cao, Tan-Yun Cheng, Dennis Dam, Adam Shahine, Martin D. Witte, Dmitri Filippov, Gijsbert A. van der Marel, D. Branch Moody, Adriaan J. Minnaard, Jamie Rossjohn, Jeroen Codee, Ildiko Van Rhijn

Materials included in supporting information:

Supporting information 1: Chemical syntheses

Figure S1: NOESY NMR Spectra

Figure S2: Hydrolysis of MPM and analogs

Figure S3: Unbiased electron density maps

Figure S4: Lack of detection of a CD1c-MPM-3 orCD1c-MPM tetramer-positive population in three healthy donors

Table S1: Data collection and refinement statistics

Supporting information 1: Synthesis of MPM analogs

List of abbreviations

AIBN	2,2′-azobis(2-methylpropionitrile)
APT	attached proton test
BnBr	benzyl bromide
CE	cyanoethyl
CSO	*(1S)*(+)-(10-camphorsulfonyl)oxaziridine
DCI	4,5-dicyanoimidazole
DCM	dichloromethane
DDQ	2,3-dichloro-5,6-dicyano-1,4-benzoquinone
DIPA	diisopropylamine
DIPEA	*N,N*-diisopropylethylamine
DMF	dimethylformamide
DMSO	dimethyl sulfoxide
DPAP	2,2-dimethoxy-2-phenylacetophenone
EtOAc	ethyl acetate
HRMS	high resolution mass spectrometry
IBX	2-iodoxybenzoic acid
KHMDS	potassium bis(trimethylsilyl)amide
LDA	lithium diisopropylamide
*m*CPBA	*meta*-chloroperoxybenzoic acid
MPM	mannosyl phosphomycoketide
NapBr	1-bromonaphthalene
NBS	*N*-bromosuccinimide
NMR	nuclear magnetic resonance
NOE	nuclear Overhauser effect
NOESY	nuclear Overhauser effect spectroscopy
PBS	phosphate-buffered saline
*p*TsOH	*para*-toluenesulfonic acid
quant.	quantitative
RT	room temperature
TBAF	tetra-*n*-butylammonium fluoride

TBAI	tetra-*n*-butylammonium iodide
TDSCl	chloro(dimethyl)thexylsilane
THF	tetrahydrofuran
TMSBr	trimethylsilyl bromide

Synthesis of *C*-mannosyl-1-phosphomycoketide (MPM-1)

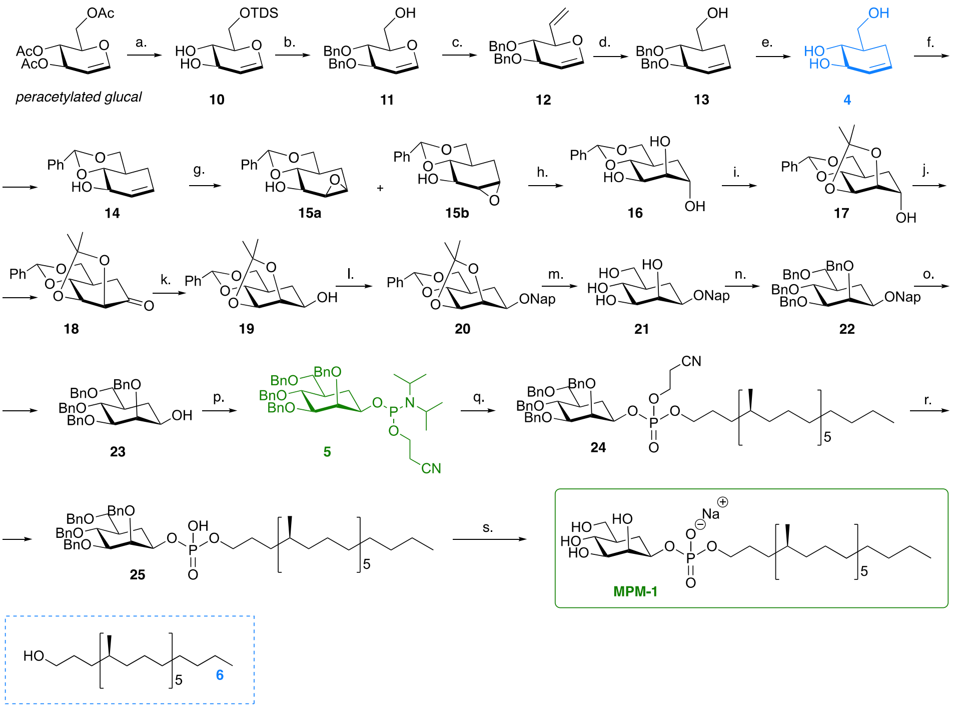

Synthetic scheme for the generation of carba-mannose (MPM-1). a) i. K_2CO_3, MeOH, ii. TDSCl, imidazole, DMF, -20°C, y: 82%, b) i. BnBr, NaH, TBAI, THF, ii. TBAF, THF, y: 69%, c) i. IBX, EtOAc, reflux, ii. PPh_3CH_3I, KHMDS, THF, -78°C to RT, y: 88%, d) i. *o*-dichlorobenzene, 230°C, ii. $NaBH_4$, EtOH/THF, y: 94%, e) Li-naphthalenide, THF, -20°C, y: 81%, f) $PhCH(OMe)_2$, pTsOH, DMF, 60°C, y: 67%, g) *m*-CPBA, PBS buffer, DCM, α-gluco y: 5%, β-manno y: 86%, h) KOH, dioxane, H_2O, 90°C, y: quant., i) 2,2-dimethoxypropane, pTsOH, DMF, y: 90%, j) IBX, EtOAc, reflux, y: 82%, k) $NaBH_4$, DCM/MeOH, 0°C, y: 77%, l) NapBr, NaH, TBAI, THF/DMF, y: 88%, m) pTsOH, MeOH, y: 92%, n) BnBr, NaH, TBAI, DMF, y: 86%, o) DDQ, DCM/H_2O, y: 73%, p) (CEO)PCl(N-*i*Pr_2), DIPEA, DCM, y: 68%, q) i. compound **6,** DCI, CH_3CN, ii. CSO, CH_3CN, y: 70%, r) Et_3N, CH_3CN, y: 74%, s) Pd/C, H_2, $CHCl_3$:MeOH (1:1 v/v), y: 47%.

6-O-dimethylthexylsilyl-D-glucal (10).

3,4,6-O-acetyl-D-glucal (81.6 g, 300 mmol, 1 eq) was dissolved in MeOH (500 ml). To the solution K_2CO_3 (4.15 g, 30 mmol, 0.1 eq) was added and the reaction mixture was stirred

for 1 hour at room temperature. Volatiles were then removed in vacuo and the crude was co-evaporated (1x) with toluene before being dissolved in dry DMF (500 ml). To this solution imidazole (68.1 g, 900 mmol, 3 eq) was added and the mixture was cooled to -20°C. TDSCl (65 ml, 330 mmol, 1.1 eq) was added dropwise via cannula and the reaction mixture was stirred overnight at -20°C. After heating up to room temperature the reaction mixture was concentrated in vacuo, dissolved in EtOAc and transferred to a separatory funnel. The organic layer was washed (4x) with water and (2x) with brine. The combined water layers were extracted (1x) with DCM and the combined organic layers were dried over $MgSO_4$, filtered and concentrated in vacuo. Compound **10** was obtained after silicagel chromatography (Pentane/EtOAc 4:1→1:1; DCM loading of crude) as a yellow oil (71 g, 246 mmol, 82%). ^{1}H NMR (300 MHz, $CDCl_3$) δ: 6.31 (dd, J = 6.1, 1.8 Hz, 1H, H-1), 4.72 (dd, J= 6.1, 2.2 Hz, 1H, H-2), 4.34 – 4.20 (m, 1H, H-3), 4.02 – 3.93 (m, 1H, H-6a), 3.93 – 3.84 (m, 1H, H-6b), 3.82 – 3.74 (m, 2H, H-4, H-5), 3.27 (bs, 1H, OH-3), 2.59 (bs, 1H, OH-2), 1.70 – 1.54 (m, 1H, CH-TDS), 0.90 – 0.84 (m, 12H, CH_3-TDS), 0.16 – 0.10 (m, 6H, CH_3-Si). ^{13}C-APT NMR (75 MHz, $CDCl_3$) δ: 144.3 (C-1), 102.6 (C-2), 76.7 (C-5), 72.7 (C-4), 69.5 (C-3), 63.8 (C-6), 34.2 (CH-TDS), 20.4 (CH_3-TDS), 18.6 (CH_3-Si).

3,4-di-O-benzyl-D-glucal (11).
Compound **10** (1.45 g, 5.0 mmol, 1 eq) was co-evaporated (3x) with toluene and dissolved in dry THF (10 mL). The solution was cooled to 0°C and TBAI (190 mg, 0.5 mmol, 0.1 eq), BnBr (2.4 mL, 20 mmol, 4 eq) and a 60% suspension in mineral oil of NaH (505 mg, 13 mmol, 2.6 eq) were added sequentially. The reaction mixture was stirred at RT overnight. Upon completion, the reaction mixture was quenched with the addition of MeOH, diluted in EtOAc and transferred to a separatory funnel. The organic layer was washed (3x) with water and (1x) with brine. The organic layer was dried over $MgSO_4$, filtered and concentrated in vacuo. The resulting crude fully protected glucal was dissolved in dry THF (10 mL) and cooled to 0°C. Then a 0.1 M solution of TBAF (70.5 mL, 7.5 mmol, 1.5 eq) in THF was added dropwise to the reaction mixture via cannula. Upon complete addition, the reaction mixture was stirred at RT overnight and subsequently quenched with the addition of a saturated solution of NH_4Cl(aq). The reaction mixture was then diluted in DCM and transferred to a separatory funnel. The organic layer was washed (1x) with brine, dried over $MgSO_4$, filtered and concentrated in vacuo. Compound **11** was obtained after silicagel chromatography (Pentane/Et_2O 9:1→1:1; DCM loading of crude) as a yellow syrup (1.13 g, 3.5 mmol, 69%). NMR analysis confirmed purity of the product, whose ^{1}H NMR and ^{13}C NMR spectra were in agreement with published literature. [1]

3,4-di-O-benzyl-6,7-ene-D-glucal (12).
Compound **11** (19.6 g, 60.3 mmol, 1 eq) was dissolved in dry EtOAc (1.2 L) after which IBX (84.6 g, 302 mmol, 5 eq) was added. The reaction mixture was stirred under reflux for 6 hours and then cooled to RT, filtered over celite and concentrated in vacuo to give the crude aldehyde. The crude aldehyde was co-evaporated (2x) with toluene and dissolved in dry THF (60 mL). A phosphonium ylide solution was then prepared by suspending PPh_3CH_3Br (43.0 g, 120.5 mmol, 2 eq) in dry THF (300 mL), cooling the suspension to -78°C and adding a 0.5 M solution of KHMDS in toluene (241 mL, 120.5 mmol, 2 eq) dropwise to the suspension via cannula. The solution was stirred for 30

minutes at -78°C and left to warm up to -50°C to obtain an intensely yellow colored solution. The phosphonium ylide solution was then cooled to -78°C and the crude aldehyde solution was added dropwise. The reaction mixture was then stirred at RT overnight. Upon completion, the reaction was quenched with the addition of a saturated solution of NH_4Cl(aq) (150 mL), then diluted in DCM and transferred to a separatory funnel. The organic layer was washed (1x) with brine and the water layer was extracted (1x) with DCM. The combined organic layers were dried over $MgSO_4$, filtered and concentrated in vacuo. Compound **12** was obtained after silicagel chromatography (Pentane/ Et_2O 39:1→3:2; DCM loading of crude) as a light brown syrup (17.1 g, 53.1 mmol, 88%). NMR analysis confirmed purity of the product, whose ^{1}H NMR and ^{13}C NMR spectra were in agreement with published literature. [2]

3,4-di-O-benzyl-pseudo-D-glucal (13).

Compound **12** (645 mg, 2 mmol, 1 eq) was co-evaporated (3x) with toluene, transferred to a 5 mL microwave vial purged with N_2 and dissolved in dry *o*-dichlorobenzene (5 mL). The microwave vial was purged once more with N_2 and stirred under microwave irradiation for 20 minutes at 230°C. The intermediate aldehyde was then reduced by pouring the reaction mixture in a solution on of $NaBH_4$ (113 mg, 3 mmol, 1.5 eq) in THF/EtOH 2:1 (5 mL). This mixture was stirred for 15 minutes and quenched with the addition of water before being transferred to a separatory funnel. The water layer was extracted (3x) with DCM. The combined organic layers were washed (1x) with brine, dried over $MgSO_4$, filtered and concentrated in vacuo. Compound **13** was obtained after silicagel chromatography (Pentane/Et_2O 9:1→1:9; DCM loading of crude) as a yellow syrup (610 mg, 1.88 mmol, 94%). NMR analysis confirmed purity of the product, whose ^{1}H NMR and ^{13}C NMR spectra were in agreement with published literature. [2]

pseudo-D-glucal (4).

Naphthalene (26.8 g, 209 mmol, 7.5 eq) and freshly cut lithium pieces (10 eq) were suspended in freshly distilled dry THF (320 mL) under an Argon atmosphere. The solution was then sonicated for 30 seconds, resulting in a dark green solution of naphthalenide radicals. Dibenzylated **13** (9.02 g, 27.8 mmol, 1 eq) was co-evaporated with toluene under argon and dissolved in freshly distilled dry THF (140 mL). The resulting solution was then added dropwise via a cannula to the lithium naphthalenide solution at -78°C and stirred for 2 days at -20°C. Upon completion, non-distilled THF was added and the reaction mixture was diluted with MeOH until the dark green color disappeared. The strongly basic solution was then neutralized using Amberlite H^+, resulting in a clear solution that was filtered and concentrated in vacuo. Compound **4** was obtained after silicagel chromatography (EtOAc/MeOH 1:0→4:1; EtOAc loading of crude) as white crystals (3.25 g, 22.5 mmol, 81%). $[\alpha]_D^{20}$= - 13.7 °(c = 0.010, MeOH). ^{1}H NMR (400 MHz, MeOD) δ: 5.79 – 5.67 (m, 1H, H-1), 5.61 – 5.49 (m, 1H, H-2), 4.11 – 4.01 (m, 1H, H-3), 3.81 (dd, J = 10.9, 4.4 Hz, 1H, H-6a), 3.68 (dd, J = 10.8, 6.1 Hz, 1H, H-6b), 3.44 (dd, J = 11.1, 7.7 Hz, 1H, H-4), 2.33 – 2.21 (m, 1H, H-7a), 2.08 – 1.96 (m, 1H, H-7b), 1.93 – 1.79 (m, 1H, H-5). ^{13}C-APT NMR (101 MHz, $CDCl_3$) δ: 130.2 (C-2), 128.3 (C-1), 76.0 (C-4), 74.7 (C-3), 64.5 (C-6), 42.5 (C-5), 29.5 (C-7). HRMS $[M+Na]^+$: 167.0682 found, 167.0679 calculated.

4,6-O-benzylidene-pseudo-D-glucal (14).

Fully deprotected pseudo-glucal **4** (3.0 g, 20.8 mmol, 1 eq) was dissolved in dry DMF (42 mL). Benzaldehyde dimethyl acetal (4.65 mL, 31.2 mmol, 1.5 eq) and pTsOH (363 mg, 0.2 mmol, 0.1 eq) were added and the flask containing the reaction mixture was spun on a rotary evaporator under reduced pressure at 60°C. After 1 hour a saturated solution of $NaHCO_3$(aq) was added and the reaction mixture was diluted in water and Et_2O and transferred to a separatory funnel. The water layer was extracted (3x) with Et_2O. The combined organic layers were washed (1x) with brine, dried over $MgSO_4$, filtered and concentrated in vacuo. Compound **14** was obtained after crystallization from EtOH as white crystals (3.24 g, 14 mmol, 67%). $[\alpha]_D^{20}$= - 11.6 °(c = 0.010, DCM). ^{1}H NMR (400 MHz, $CDCl_3$) δ: 7.58 – 7.48 (m, 2H, H-arom), 7.44 – 7.33 (m, 3H, H-arom), 5.74 (ddd, J = 8.9, 4.5, 2.2 Hz, 1H, H-1), 5.69 – 5.59 (m, 2H, H-2, CH-Ph), 4.48 – 4.38 (m, 1H, H-3), 4.22 (dd, J = 11.2, 4.8 Hz, 1H, H-6a), 3.75 – 3.62 (m, 2H, H-4, H-6b), 2.24 – 2.04 (m, 2H, H-5, H-7a), 1.84 – 1.77 (m, 1H, H-7b). ^{13}C-APT NMR (101 MHz, $CDCl_3$) δ: 138.2 (C-arom), 129.2 (C-arom), 128.6 (C-arom), 128.5 (C-2), 126.8 (C-1), 126.3 (C-arom), 101.8 (CH-Ph), 83.5 (C-4), 71.3 (C-6), 70.5 (C-3), 34.0 (C-5), 26.6 (C-7). HRMS $[M+Na]^+$: 255.0992 found, 255.0992 calculated.

1,2-oxirane-4,6-O-benzylidene-pseudo-D-pyranoside (15a & 15b).

Benzylidene protected pseudo-glucal **14** (2.5 g, 8.7 mmol, 1 eq) was dissolved in an emulsion of DCM (29 mL) and PBS (10.5 mL). Then *m*CPBA (4.0 g, 17.4 mmol, 2 eq) was added in various portions to the emulsion. After stirring for 2 hours, the reaction mixture was diluted in DCM and transferred to a separatory funnel. The organic layer was washed (1x) with water, (1x) with a saturated solution of $Na_2S_2O_3$(aq), (1x) with water and (1x) with brine. The organic layer was then dried over $MgSO_4$, filtered and concentrated in vacuo. Compound **15a** was obtained after silicagel chromatography (Pentane/ Et_2O 7:3→0:1; DCM loading of crude) as a white solid (1.9 g, 7.5 mmol, 86%).

β-manno: $[\alpha]_D^{20}$= - 0.4 °(c = 0.010, DCM). ^{1}H NMR (400 MHz, $CDCl_3$) δ: 7.51 – 7.44 (m, 2H, H-arom), 7.42 – 7.32 (m, 3H, H-arom), 5.49 (s, 1H, CH-Ph), 4.19 – 4.07 (m, 2H, H-6a, H-3), 3.72 (dd, J = 10.6, 8.4 Hz, 1H, H-4), 3.52 (pt, J = 10.9 Hz, 1H, H-6b), 3.41 (dd, J = 4.0, 2.0 Hz, 1H, H-2), 3.34 (dd, J = 4.8, 3.9 Hz, 1H, H-1), 2.55 (s, 1H, OH), 2.05 – 1.85 (m, 2H, H-7a, H-5), 1.63 – 1.52 (m, 2H, H-7b). ^{13}C-APT NMR (101 MHz, $CDCl_3$) δ: 138.0 (C-arom), 129.3 (C- arom), 128.5 (C-arom), 126.3 (C-arom), 101.7 (CH-Ph), 79.9 (C-4), 71.6 (C-3), 70.8 (C-6), 56.1 (C-2), 52.9 (C-1), 34.2 (C-5), 24.8 (C-7). HRMS $[M+Na]^+$: 271.0939 found, 271.0941 calculated.

The epoxide **15b** was also isolated as a white solid (108 mg, 0.44 mmol, 5%).

α-gluco: $[\alpha]_D^{20}$= + 1.6 °(c = 0.010, DCM). ^{1}H NMR (400 MHz, $CDCl_3$) δ: 7.50 – 7.43 (m, 2H, H-arom), 7.40 – 7.31 (m, 3H, H-arom), 5.45 (s, 1H, CH-Ph), 4.12 (dd, J = 11.2, 4.8 Hz, 1H, H-6a), 3.88 (dd, J = 8.2, 0.7 Hz, 1H, H-3), 3.47 (pt, J = 11.2 Hz, 1H, H-6b), 3.35 (dd, J = 10.9, 8.2 Hz, 1H, H-4), 3.18 (dt, J = 3.7, 1.9 Hz, 1H, H-1), 3.04 (pd, J = 3.5 Hz, 1H, H-2), 2.01 (ddd, J = 14.7, 4.6, 2.0 Hz, 1H, H-7a), 1.91 – 1.76 (m, 1H, H-5), 1.38 (ddd, J = 14.8, 11.9, 1.7 Hz, 1H, H-7b). ^{13}C-APT NMR (101 MHz, $CDCl_3$) δ: 137.8 (C-arom), 129.2 (C-arom), 128.4 (C-arom), 126.3 (C-arom), 101.6 (CH-Ph), 82.7 (C-4), 71.2 (C-6), 69.3 (C-3), 55.7 (C-2), 52.0 (C-1), 27.4 (C-5), 25.5 (C-7). HRMS $[M+Na]^+$: 271.0944 found, 271.0941 calculated.

4,6-O-benzylidene-7-carba-α-D-mannopyranoside (16).

A mixture of the epoxides **15a** and **15b** (745 mg, 3.0 mmol, 1 eq) was dissolved in dioxane (6 mL) and a 5M solution of KOH (54 mL) was added. The reaction mixture was stirred at 90°C for 2 hours and 30 minutes and upon completion cooled to 0°C, diluted in water and transferred to a separatory funnel. The water layer was extracted (3x) with EtOAc and the combined organic layers were washed (1x) with brine, dried over $MgSO_4$, filtered and concentrated in vacuo. Compound **16** was obtained as a white solid (796 mg, 3 mmol, quant.) without any further purification. $[\alpha]_D^{20}$= - 44.7 °(c = 0.010, MeOH). ^{1}H NMR (400 MHz, MeOD) δ: 7.62 – 7.48 (m, 2H, H-arom), 7.48 – 7.30 (m, 3H, H-arom), 5.64 (s, 1H, CH-Ph), 4.10 (dd, J = 10.9, 4.4 Hz, 1H, H-6a), 4.02 – 3.96 (m, 2H, H-1, H-2), 3.94 (dd, J = 9.6, 2.8 Hz, 1H, H-3), 3.86 (pt, J = 9.8 Hz, 1H, H-4), 3.70 (t, J = 11.0 Hz, 1H, H-6b), 2.32 – 2.11 (m, 1H, H-5), 1.62 (td, J = 13.4, 2.4 Hz, 1H, H-7a), 1.55 – 1.41 (m, 1H, H-7b). ^{13}C-APT NMR (101 MHz, $CDCl_3$) δ: 140.1 (C-arom), 129.7 (C-arom), 129.0 (C-arom), 127.5 (C-arom), 103.3 (CH-Ph), 82.2 (C-4), 74.8 (C-3), 72.4 (C-6), 71.1 (C-2), 70.7 (C-1), 34.1 (C-5), 28.4 (C-7). HRMS $[M+Na]^+$: 289.1045 found, 289.1046 calculated.

2,3-O-isopropylidene-4,6-O-benzylidene-7-carba-α-D-mannopyranoside (17).

Benzylidene protected pseudo-mannoside **16** (591 mg, 2.2 mmol, 1 eq) was dissolved in dry DMF (22 mL). The solution was then cooled to 0°C and 2,2-dimethoxypropane (1.1 mL, 8.8 mmol, 4 eq) and *p*TsOH (42 mg, 0.22 mmol, 0.1 eq) were added. The reaction mixture was left to stir at RT overnight. Then the reaction was quenched with Et_3N, diluted in water and transferred to a separatory funnel. The water layer was extracted (3x) with Et_2O. The combined organic layers were washed (1x) with brine, dried over $MgSO_4$, filtered and concentrated in vacuo. Compound **17** was obtained after silicagel chromatography (Pentane/Et_2O 9:1→0:1; DCM loading of crude; silica was neutralized with Et_3N) as a white solid (606 mg, 1.98 mmol, 90%). Note: this compound readily degrades in non-neutralized $CDCl_3$. $[\alpha]_D^{20}$= -51.6 °(c = 0.010, DCM). ^{1}H NMR (400 MHz, $CDCl_3$) δ: 7.56 – 7.45 (m, 2H, H-arom), 7.42 – 7.28 (m, 3H, H-arom), 5.55 (s, 1H, CH-Ph), 4.32 (dd, J = 7.8, 5.3 Hz, 1H, H-3), 4.24 – 4.15 (m, 3H, H-1, H-2, H-6a), 3.69 (dd, J = 11.2, 7.7 Hz, 1H, H-4), 3.61 (pt, J = 11.0 Hz, 1H, H-6b), 2.26 – 2.11 (m, 1H, H-5), 1.87 (s, 1H, OH), 1.64 – 1.41 (m, 5H, H-7, CH_3-isopr), 1.38 (s, 3H, CH_3-isopr). ^{13}C-APT NMR (101 MHz, $CDCl_3$) δ: 138.1 (C-arom), 129.0 (C-arom), 128.3 (C-arom), 126.5 (C-arom), 109.6 (C-isopr), 101.9 (CH-Ph), 82.3 (C-4), 78.8 (C-2), 76.9 (C-3), 71.5 (C-6), 67.2 (C-1), 30.3 (C-5), 28.6 (C-7), 28.3 (CH_3-isopr), 26.0 (CH_3-isopr). HRMS $[M+Na]^+$: 329.1353 found, 329.1359 calculated.

1-one-2,3-O-isopropylidene-4,6-O-benzylidene-7-carba-D-mannopyranoside (18).

Acetal protected compound **17** (319 mg, 1.0 mmol, 1 eq) was dissolved in dry EtOAc (20 mL). IBX (1.4 g, 5.0 mmol, 5 eq) was added to the solution and the reaction mixture was refluxed overnight. The reaction mixture was then cooled to RT, filtered over celite and concentrated in vacuo. Compound **18** was obtained after silicagel chromatography (Pentane/EtOAc 9:1→1:1; DCM loading of crude; silica was neutralized with Et_3N) as a white solid (250 mg, 0.82 mmol, 82%). Note: this compound readily degrades in non-neutralized $CDCl_3$. $[\alpha]_D^{20}$= -3.2 °(c = 0.010, DCM). ^{1}H NMR (400 MHz, $CDCl_3$) δ: 7.56 – 7.49 (m, 2H, H-arom), 7.42 – 7.31 (m, 3H, H-arom), 5.57 (s, 1H, CH-Ph), 4.73 (dd, J = 8.5, 6.7 Hz, 1H, H-2), 4.63 (d, J = 8.6 Hz, 1H, H-3), 4.33 (dd, J = 11.3, 4.8 Hz, 1H, H-6a), 3.76 (dd, J

= 11.3, 6.8 Hz, 1H, H-4), 3.61 (dd, J = 11.4, 10.4 Hz, 1H, H-6b), 2.56 (ddd, J = 17.6, 6.9, 1.0 Hz, 1H, H-7a), 2.52 – 2.40 (m, 1H, H-5), 2.03 (dd, J = 17.5, 11.0 Hz, 1H, H-7b), 1.54 (s, 3H, CH_3-isopr), 1.40 (s, 3H, CH_3-isopr). ^{13}C-APT NMR (101 MHz, $CDCl_3$) δ: 204.7 (C-1), 137.5 (C-arom), 129.4 (C-arom), 128.4 (C-arom), 126.4 (C-arom), 111.9 (C-isopr), 101.6 (CH-Ph), 80.7 (C-4), 79.3 (C-3), 78.5 (C-2), 71.3 (C-6), 36.7 (C-7), 30.8 (C-5), 27.2 (CH_3-isopr), 25.3 (CH_3-isopr). HRMS $[M+Na]^+$: 327.1204 found, 327.1203 calculated.

2,3-O-isopropylidene-4,6-O-benzylidene-7-carba-β-D-mannopyranoside (19).

Ketone **18** (250 mg, 0.82 mmol, 1 eq) was dissolved in a mixture of DCM/MeOH 20:1 (16.5 mL). The solution was cooled to 0°C and $NaBH_4$ (155 mg, 4.1 mmol, 5 eq) was added in portions. After stirring for 30 minutes the reaction was quenched with the addition of water and transferred to a separatory funnel. The water layer was extracted (3x) with DCM and the combined organic layers were washed (1x) with brine, dried over $MgSO_4$, filtered and concentrated in vacuo. Compound **19** was obtained after silicagel chromatography (Pentane/EtOAc 7:3→3:7; DCM loading of crude; silica was neutralized with Et_3N) as a white solid (193 mg, 0.63 mmol, 77%) with an axial to equatorial ratio of 1:32. Note: this compound readily degrades in non-neutralized $CDCl_3$. $[\alpha]_D^{20}$= -70.7 °(c = 0.010, DCM). ^1H NMR (400 MHz, $CDCl_3$) δ: 7.54 – 7.46 (m, 2H, H-arom), 7.38 – 7.30 (m, 3H, H-arom), 5.55 (s, 1H, CH-Ph), 4.39 (dd, J = 6.0, 3.8 Hz, 1H, H-2), 4.25 – 4.15 (m, 2H, H-3, H-6a), 4.06 – 3.96 (m, 1H, H-1), 3.84 (dd, J = 10.7, 7.6 Hz, 1H, H-4), 3.65 (pt, J = 10.8 Hz, 1H, H-6b), 2.29 (d, J = 6.6 Hz, 1H, OH), 1.81 – 1.66 (m, 2H, H-7a, H-5), 1.59 (s, 3H, CH_3-isopr), 1.46 – 1.34 (m, 4H, CH_3-isopr, H-7b). ^{13}C-APT NMR (101 MHz, $CDCl_3$) δ: 138.1 (C-arom), 129.0 (C-arom), 128.3 (C-arom), 126.4 (C-arom), 109.8 (C-isopr), 101.9 (CH-Ph), 81.9 (C-4), 77.9 (C-3), 76.6 (C-2), 71.4 (C-6), 67.7 (C-1), 32.5 (C-5), 28.6 (C-7), 28.0 (CH_3-isopr), 25.7 (CH_3-isopr). HRMS $[M+Na]^+$: 329.1360 found, 329.1359 calculated.

1-O-naphthyl-2,3-O-isopropylidene-4,6-O-benzylidene-7-carba-β-D-mannopyranoside (20).

Acetal protected pseudo-mannoside **19** (2.23 g, 7.28 mmol, 1 eq) was co-evaporated with toluene and dissolved in dry DMF (38 mL). The solution was cooled to 0°C and TBAI (269 mg, 0.728 mmol, 0.1 eq) and naphthyl bromide (3.22 g, 14.6 mmol, 2 eq) were added. Then a 60% suspension in mineral oil of NaH (43.7 mg, 10.9 mmol, 1.5 eq) was added in multiple portions to the reaction mixture. After the addition of NaH was complete, the solution was stirred at RT for 2 hours and then quenched with the addition of MeOH. The reaction mixture was then diluted in Et_2O and water and transferred to a separatory funnel. The water layer was extracted (3x) with Et_2O and the combined organic layers were washed (1x) with brine, dried over $MgSO_4$, filtered and concentrated in vacuo. Compound **20** was obtained after silicagel chromatography (Pentane/EtOAc 4:1→0:1; DCM loading of crude; silica was neutralized with Et_3N) as a white solid (2.85 g, 6.41 mmol, 88%). Note: this compound readily degrades in non-neutralized $CDCl_3$. $[\alpha]_D^{20}$= -35.5 °(c = 0.010, DCM). ^1H NMR (400 MHz, $CDCl_3$) δ: 7.89 – 7.77 (m, 4H, H-arom), 7.56 – 7.43 (m, 5H, H-arom), 7.36 – 7.26 (m, 3H, H-arom), 5.52 (s, 1H, CH-Ph), 4.92 – 4.78 (m, 2H, CH_2-Nap), 4.51 – 4.42 (m, 1H, H-2), 4.15 (dd, J = 11.0, 4.4 Hz, 1H, H-6a), 4.08 (dd, J = 7.8, 5.0 Hz, 1H, H-3), 3.81 – 3.72 (m, 1H, H-1), 3.72 – 3.59 (m, 2H, H-4, H-6b), 1.77 – 1.67 (m, 1H, H-7), 1.67 – 1.45 (m, 5H, CH_3-isopr, H-5, H-7), 1.41 (s, 3H, CH_3-isopr). ^{13}C-APT NMR

(101 MHz, $CDCl_3$) δ: 138.0 (C-arom), 135.6 (C-arom), 133.3 (C-arom), 133.2 (C-arom), 129.0 (C-arom), 128.5 (C-arom), 128.2 (C-arom), 128.0 (C-arom), 127.9 (C-arom), 126.8 (C-arom), 126.4 (C-arom), 126.4 (C-arom), 126.2 (C-arom), 125.9 (C-arom), 110.1 (C-isopr), 102.0 (CH-Ph), 82.8 (C-4), 78.2 (C-3), 75.3 (C-2), 74.0 (C-1), 71.3 (CH_2-Nap), 71.1 (C-6), 33.3 (C-5), 28.7 (CH_3-isopr), 26.4 (CH_3-isopr), 26.0 (C-7). HRMS $[M+Na]^+$: 469.1983 found, 469.1986 calculated.

1-O-naphthyl-7-carba-β-D-mannopyranoside (21).

The naphthyl protected **20** (89.3 mg, 0.2 mmol, 1 eq) was dissolved in DCM/MeOH 1:1 (5 mL) and *p*TsOH (11.4 mg, 0.06 mmol, 0.3 eq) was added. The reaction mixture was stirred at RT for 2 hours, then quenched with the addition of Et_3N and concentrated in vacuo. Compound **21** was obtained after silicagel chromatography (EtOAc/MeOH 1:0→4:1; EtOAc loading of crude) as a white solid (58.5 mg, 0.184 mmol, 92%). $[\alpha]_D^{20}$= + 20.5 °(c = 0.010, MeOH). ^{1}H NMR (400 MHz, MeOD) δ: 7.91 – 7.82 (m, 4H, H-arom), 7.58 – 7.44 (m, 3H, H-arom), 4.86 – 4.72 (m, 2H, CH_2-Nap), 4.30 – 4.19 (m, 1H, H-2), 3.84 (dd, J = 10.7, 4.3 Hz, 1H, H-6a), 3.64 – 3.50 (m, 3H, H-1, H-6, H-4), 3.30 (dd, J = 9.4, 2.8 Hz, 1H, H-3), 2.03 – 1.87 (m, 1H, H-7a), 1.73 (pq, J = 12.5 Hz, 1H, H-7b), 1.53 – 1.38 (m, 1H, H-5). ^{13}C-APT NMR (101 MHz, MeOD) δ: 137.5 (C-arom), 134.8 (C-arom), 134.5 (C-arom), 129.1 (C-arom), 128.9 (C-arom), 128.7 (C-arom), 127.5 (C-arom), 127.1 (C-arom), 126.9 (C-arom), 126.9 (C-arom), 78.1 (C-4), 76.4 (C-3), 72.3 (C-1), 72.0 (C-2), 71.4 (CH_2-Nap), 64.8 (C-6), 42.5 (C-5), 28.3 (C-7). HRMS $[M+Na]^+$: 341.1358 found, 341.1359 calculated.

1-O-naphthyl-2,3,4,6-tetra-O-benzyl-7-carba-β-D- mannopyranoside (22).

Naphthyl protected carba-mannoside **21** (726.4 mg, 2.28, 1 eq) was co-evaporated with toluene and dissolved in dry DMF (25 mL). The solution was cooled to 0°C and benzyl bromide (2.171 mL, 18.25 mmol, 8 eq) and TBAI (84 mg, 0.228 mmol, 0.1 eq) were added. Then a 60% suspension in mineral oil of NaH (456 mg, 11.41 mmol, 5 eq) was added in multiple portions to the reaction mixture. After the addition of NaH was complete, the solution was stirred at RT overnight and then quenched with the addition of MeOH. The reaction mixture was then diluted in Et_2O and water and transferred to a separatory funnel. The water layer was extracted (3x) with Et_2O and the combined organic layers were washed (1x) with brine, dried over $MgSO_4$, filtered and concentrated in vacuo. Compound **22** was obtained after silicagel chromatography (Pentane/Et_2O 20:1→4:1; DCM loading of crude) as a pale syrup (1.36 g, 1.96 mmol, 86%). ^{1}H NMR (400 MHz, $CDCl_3$) δ: 7.86 – 7.72 (m, 4H, H-arom), 7.52 – 7.39 (m, 5H, H-arom), 7.37 – 7.17 (m, 19H, H-arom), 4.96 – 4.88 (m, 3H, CH_2-Nap, CH_2-Bn), 4.70 (d, J = 12.2 Hz, 1H, CH_2-Bn), 4.66 – 4.59 (m, 3H, CH_2-Bn), 4.53 – 4.46 (m, 3H, CH_2-Bn), 4.21 – 4.14 (m, 1H, H-2), 3.83 (dd, J = 10.6, 9.3 Hz, 1H, H-4), 3.64 (dd, J = 8.8, 3.0 Hz, 1H, H-6a), 3.48 (dd, J = 8.8, 6.9 Hz, 1H, H-6b), 3.43 (ddd, J = 11.2, 5.1, 2.1 Hz, 1H, H-1), 3.37 (dd, J = 9.4, 2.4 Hz, 1H, H-3), 2.15 – 1.99 (m, 2H, H-7), 1.74 – 1.61 (m, 1H, H-5). ^{13}C-APT NMR (101 MHz, $CDCl_3$) δ: 139.7 (C-arom), 139.0 (C-arom), 138.8 (C-arom), 138.7 (C-arom), 136.3 (C-arom), 133.4 (C-arom), 133.0 (C-arom), 128.5 (C-arom), 128.4 (C-arom), 128.2 (C-arom), 128.2 (C-arom), 128.0 (C-arom), 127.9 (C-arom), 127.8 (C-arom), 127.7 (C-arom), 127.7 (C-arom), 127.6 (C-arom), 127.6 (C-arom), 127.4 (C-arom), 127.3 (C-arom), 126.2 (C-arom), 126.0 (C-arom), 125.9 (C-arom), 125.6 (C-arom), 84.5 (C-3), 78.4 (C-4), 78.2 (C-1), 75.6 (C-2), 75.4 (CH_2-Bn), 73.8

(CH_2-Bn), 73.2 (CH_2-Bn), 72.3 (CH_2-Bn), 71.1 (C-6), 70.9 (CH_2-Nap), 39.9 (C-5), 28.4 (C-7). HRMS $[M+Na]^+$: 701.3229 found, 701.3238 calculated.

2,3,4,6-tetra-O-benzyl-7-carba-β-D-mannopyranoside (23).

Fully protected carba-mannoside **22** (505 mg, 0.744 mmol, 1 eq) was dissolved in an emulsion of DCM/water 9:1 (7.5 mL). The solution was stirred in the dark and then DDQ (169 mg, 0.744 mmol, 1 eq) was added. After 2 hours the reaction mixture was diluted with DCM and transferred to a separatory funnel. The organic layer was washed (1x) with (1x) a saturated solution of $NaHCO_3$(aq), (1x) with a saturated solution of $Na_2S_2O_3$(aq), and again (1x) a saturated solution of $NaHCO_3$(aq) until both the organic and the water layer turned clear. The organic layer was then washed (1x) with brine, dried over $MgSO_4$, filtered and concentrated in vacuo. Compound **23** was obtained after silicagel chromatography (Pentane/Et_2O 4:1→3:7; DCM loading of crude) as a light-yellow solid (292 mg, 0.542 mmol, 73%). $[\alpha]_D^{20}$= + 10.2 °(c = 0.010, DCM). ^1H NMR (400 MHz, $CDCl_3$) δ: 7.43 – 7.19 (m, 20H, H-arom), 5.16 (d, J = 11.7 Hz, 1H, CH_2-Bn), 4.89 (d, J = 10.8 Hz, 1H, CH_2-Bn), 4.75 (s, 2H, CH_2-Bn), 4.65 (d, J = 11.6 Hz, 1H, CH_2-Ph), 4.52 (d, J = 10.9 Hz, 1H, CH_2-Ph), 4.47 (d, J = 3.1 Hz, 2H, CH_2-Ph), 4.05 – 3.98 (m, 1H, H-2), 3.82 (pt, J = 9.8 Hz, 1H, H-4), 3.62 – 3.50 (m, 3H, H-1, H-6), 3.47 (dd, J = 9.4, 2.3 Hz, 1H, H-3), 2.02 (s, 1H, OH), 1.94 – 1.84 (m, 1H, H-7a), 1.78 (pq, J = 12.1 Hz, 1H, H-7b), 1.72 – 1.61 (m, 1H, H-5). ^{13}C-APT NMR (101 MHz, $CDCl_3$) δ: 139.2 (C-arom), 138.9 (C-arom), 138.7 (C-arom), 138.6 (C-arom), 128.6 (C-arom), 128.6 (C-arom), 128.5 (C-arom), 128.2 (C-arom), 128.0 (C-arom), 127.8 (C-arom), 127.8 (C-arom), 127.7 (C-arom), 127.7 (C-arom), 127.6 (C-arom), 127.6 (C-arom), 85.0 (C-3), 79.4 (C-2), 78.0 (C-4), 75.3 (CH_2-Bn), 74.6 (CH_2-Bn), 73.1 (CH_2-Bn), 73.0 (CH_2-Bn), 70.6 (C-6), 69.6 (C-1), 39.5 (C-5), 32.2 (C-7). HRMS $[M+Na]^+$: 561.2607 found, 561.2612 calculated.

1-O-([N,N-diisopropylamino]-2-O-benzyl-phosphite)-2,3,4,6-tetra-O-benzyl-7-carba-β-D-mannopyranoside (5).

Carba-mannoside **23** (182.4 mg, 0.339 mmol, 1eq) was co-evaporated (2x) with toluene, dissolved in dry DCM (2 ml). DIPEA (0.089 mL, 0.51 mmol, 1.5 eq) and activated 4Å molecular sieves were added and the solution was stirred for 15 minutes under an Argon atmosphere. Then 2-cyanoethyl *N,N*-diisopropylchlorophosphoramidite (0.406 mmol, 0.091 mL, 1.2 eq) was added to the solution. After stirring for 45 minutes, the reaction was quenched with the addition of water. The following workup and purification were performed as quickly as possible: the reaction mixture was diluted in DCM and transferred to a separatory funnel. The organic layer was washed (1x) with a mixture containing a saturated solution of $NaHCO_3$(aq) and brine 1:1. The organic layer was then dried over Na_2SO_4, filtered and concentrated in vacuo. Compound **5** was obtained after silicagel chromatography (Pentane/Et_2O 9:1→1:1; DCM loading of crude; silica was neutralized with Et_3N) as a colorless syrup (170 mg, 0.229 mmol, 68%). ^1H NMR (500 MHz, CD_3CN) δ: 7.59 – 7.07 (m, 20H, H-arom), 4.99 – 4.79 (m, 3H, CH_2-Bn), 4.78 – 4.68 (m, 1H, CH_2-Bn), 4.67 – 4.59 (m, 1H, CH_2-Bn), 4.53 – 4.41 (m, 3H, CH_2-Bn), 4.20 (pd, J = 70.87 Hz, 1H, H-2), 4.01 – 3.87 (m, 1H, H-1), 3.87 – 3.59 (m, 5H, CH-N, H-4, OCH_2), 3.59 – 3.48 (m, 3H, H-3, H-6), 2.73 – 2.59 (m, 2H, CH_2CN), 2.05 (pq, J = 12.5 Hz, 1H, H-7a), 1.99 – 1.81 (m,

1H, H-7b), 1.73 – 1.61 (m, 1H, H-5), 1.28 – 1.11 (m, 12H, CH_3). ^{31}P-NMR (202 MHz, CD_3CN) δ: 147.49, 148.66.

2,3,4,6-tetra-O-benzyl-7-carba-β-D-mannopyranosyl-1-(2-O-cyanoethylphosphate)-(4S,8S,12S,16S,20S)-4,8,12,16,20-pentamethylheptacosyl (24).

Lipid **6** (46.3 mg, 0.099 mmol, 1 eq) was co-evaporated (3x) with toluene and then dissolved in a 0.25 M solution of DCI in acetonitrile (0.60 mL, 0.15 mmol, 1.5 eq). Then an additional portion of dry acetonitrile (0.6 mL) was added to dissolve the lipid. 4Å Molecular sieves were added and the solution was stirred for 15 minutes under an Argon atmosphere. A 0.1 M solution of phosphoramidite **5** (2.2 mL, 0.22 mmol, 2.2 eq) in dry acetonitrile was then added slowly to the reaction mixture. The reaction mixture was stirred for 3 hours and upon complete coupling, a 0.25 M solution of CSO (1.2 mL, 0.31 mmol, 3 eq) in dry acetonitrile was added to the reaction mixture. After stirring for 15 minutes, water was added and the reaction mixture was diluted in EtOAc. The organic layer was washed with a mixture containing a saturated solution of $NaHCO_3$(aq) and brine 1:1 and the water layer was extracted (2x) with EtOAc. The combined organic layers were dried over Na_2SO_4, filtered and concentrated in vacuo. Compound **24** was obtained after silicagel chromatography (Pentane/EtOAc 5:1→1:1; DCM loading of crude; silica was neutralized with Et_3N) followed by size exclusion (LH-20, DCM/MeOH, 1/1, v/v) as a colorless syrup (78 mg, 0.070 mmol, 70%). $[\alpha]_D^{20}$= + 1.2 °(c = 0.005, DCM). ^{1}H NMR (400 MHz, $CDCl_3$) δ: 7.45 – 7.38 (m, 2H, H-arom), 7.36 – 7.25 (m, 16H, H-arom), 7.22 – 7.16 (m, 2H, H-arom), 5.03 – 4.92 (m, 1H, CH_2-Bn), 4.92 – 4.84 (m, 1H, CH_2-Bn), 4.80 (d, J = 12.1 Hz, 1H, CH_2-Bn), 4.74 – 4.61 (m, 2H, CH_2-Bn), 4.53 – 4.42 (m, 3H, CH_2-Bn), 4.42 – 4.33 (m, 1H, H-1), 4.26 – 4.18 (m, 1H, H-2), 4.18 – 4.10 (m, 1H, OCH_2-lipid), 4.10 – 3.95 (m, 3H, OCH_2-lipid, CH-N), 3.90 – 3.78 (m, 1H, H-4), 3.61 – 3.50 (m, 2H, H-6), 3.49 – 3.41 (m, 1H, H-3), 2.67 (t, J = 6.3 Hz, 1H, CH_2CN), 2.61 (td, J = 6.3, 2.7 Hz, 1H, CH_2CN), 2.29 – 2.14 (m, 1H, H-7a), 2.07 – 1.95 (m, 1H, H-7b), 1.76 – 1.55 (m, 3H, H-5, CH_2-lipid), 1.44 – 0.97 (m, 45H, CH_2-lipid, CH-lipid), 0.92 – 0.78 (m, 18H, CH_3-lipid). ^{13}C-APT NMR (101 MHz, $CDCl_3$) δ: 139.3 (C-arom), 139.2 (C-arom), 138.8 (C-arom), 138.6 (C-arom), 138.5 (C-arom), 138.5 (C-arom), 128.5 (C-arom), 128.4 (C-arom), 128.3 (C-arom), 128.2 (C-arom), 127.7 (C-arom), 127.7 (C-arom), 127.7 (C-arom), 127.6 (C-arom), 127.6 (C-arom), 127.6 (C-arom), 127.5 (C-arom), 127.5 (C-arom), 127.4 (C-arom), 127.4 (C-arom), 116.5 (CN), 116.4 (CN'), 84.0 (C-3), 77.6, 77.6, 77.5, 77.5, 77.4, 77.4, 77.3, 77.2, 77.1, 75.4 (CH_2-Bn), 74.6 (CH_2-Bn), 74.5 (CH_2-Bn'), 73.1 (CH_2-Bn), 72.6 (CH_2-Bn), 72.6 (CH_2-Bn'), 70.3 (C-6), 69.1 (d, J = 2.69 Hz, OCH_2-lipid), 69.0 (d, J = 2.61 Hz, OCH_2'-lipid), 61.7 (pt, J = 5.56 Hz, OCH_2-cyanoethyl), 39.2 (CH_2-lipid), 37.6, 37.5, 37.4, 37.2, 32.9 (CH-lipid), 32.9 (CH-lipid), 32.9 (CH-lipid), 32.7, 32.6 (CH-lipid), 32.6 (CH-lipid), 32.1, 30.1, 29.6, 29.5, 28.01 (d, J = 3.50 Hz, C-10, one diastereoisomer), 27.9 (d, J = 3.16 Hz, C-10, one diastereoisomer), 27.2, 24.6, 24.6, 22.8, 19.9 (CH_3-lipid), 19.9 (CH_3-lipid), 19.9 (CH_3-lipid), 19.7 (d, J = 1.15 Hz, CH_2CN), 19.7 (d, J = 1.36 Hz, CH_2CN'), 19.6 (CH_3-lipid), 19.6 (CH_3-lipid), 14.3 (CH_3-lipid). ^{31}P-NMR (162 MHz, $CDCl_3$) δ: -1.70, -1.65. HRMS $[M+Na]^+$: 1142.7545 found, 1142.7548 calculated.

Sodium 2,3,4,6-tetra-O-benzyl-7-carba-β-D-mannopyranosyl-1-phosphoryl-(4S,8S,12S,16S,20S)-4,8,12,16,20-pentamethylheptacosyl (25).

Cyanoethyl protected **24** (36.1 mg, 0.031 mmol, 1 eq) was co-evaporated (2x) with toluene and dissolved in dry acetonitrile (3 mL). The reaction was cooled to 0°C, then Et_3N (0.36 mL, 2.6 mmol, 80 eq) was added and the reaction was stirred at RT for 5 days. Upon completion, the reaction mixture was diluted in dry distilled toluene and concentrated. Purification by size exclusion chromatography (LH-20, DCM/MeOH, 1/1, v/v) yielded the triethylammonium salt of the product. The triethylammonium salt was converted to the sodium salt by dissolving it in MeOH and passing it through a small reaction syringe containing amberlite Na^+. Compound **25** was obtained after concentration of the eluate as a colorless oil (25,8 mg, 0.024 mmol, 74%). $[\alpha]_D^{20}$= - 1.4 °(c = 0.008, DCM). ^{1}H NMR (400 MHz, $CDCl_3$) δ: 7.47 – 7.37 (m, 2H, H-arom), 7.35 – 7.20 (m, 16H, H-arom), 7.20 – 7.13 (m, 2H, H-arom), 4.92 – 4.80 (m, 3H, CH_2-Bn), 4.58 (pq, J = 11.7 Hz, 2H, CH_2-Bn), 4.49 – 4.36 (m, 3H, CH_2-Bn), 4.34 – 4.25 (m, 1H, H-1), 4.25 – 4.19 (m, 1H, H-2), 3.93 (pq, J = 6.7 Hz, 2H, OCH_2-lipid), 3.87 – 3.75 (m, 1H, H-4), 3.58 – 3.45 (m, 2H, H-6), 3.40 (dd, J = 9.4, 2.4 Hz, 1H, H-3), 2.20 (pq, J = 12.6 Hz, 1H, H-7a), 2.04 (dt, J = 12.7, 4.5 Hz, 1H, H-7b), 1.73 – 1.48 (m, 3H, H-5, CH_2-lipid), 1.46 – 0.95 (m, 45H, CH_2-lipid, CH-lipid), 0.93 – 0.75 (m, 18H, CH_3-lipid). ^{13}C-APT NMR (101 MHz, $CDCl_3$) δ: 139.3 (C-arom), 138.9 (C-arom), 138.6 (C-arom), 138.6 (C-arom), 128.5 (C-arom), 128.5 (C-arom), 128.4 (C-arom), 128.3 (C-arom), 128.2 (C-arom), 127.7 (C-arom), 127.6 (C-arom), 127.6 (C-arom), 127.4 (C-arom), 84.0 (C-3), 77.2 (C-4), 77.2 (C-2), 76.9 (C-1), 75.3 (CH_2-Bn), 74.7 (CH_2- Bn), 73.1 (CH_2-Bn), 72.3 (CH_2-Bn), 70.4 (C-6), 68.3 (d, J = 5.46 Hz, OCH_2-lipid), 39.3 (C-5), 37.6 (CH_2-lipid), 37.6 (CH_2-lipid), 37.5 (CH_2-lipid), 37.2 (CH_2-lipid), 33.0 (CH-lipid), 33.0 (CH-lipid), 33.0 (CH-lipid), 33.0 (CH-lipid), 32.9 (CH-lipid), 32.9 (CH_2-lipid), 32.7 (CH-lipid), 32.1 (CH_2-lipid), 30.2 (CH_2-lipid), 29.9 (CH_2-lipid), 29.6 (C-7), 28.0 (d, J = 7.42 Hz, CH_2-lipid), 27.2 (CH_2-lipid), 24.6 (CH_2-lipid), 24.6 (CH_2-lipid), 22.9 (CH_2-lipid), 20.0 (CH_3-lipid), 19.9 (CH_3-lipid), 19.9 (CH_3-lipid), 19.6 (CH_3-lipid), 14.3 (CH_3-lipid). ^{31}P-NMR (162 MHz, $CDCl_3$) δ: 1.04. HRMS $[M+Na]^+$: 1088.7206 found, 1088.7205 calculated.

Sodium 7-carba-β-D-mannopyranosyl-1-phosphoryl-(4S,8S,12S,16S,20S)-4,8,12,16,20-pentamethylheptacosyl (MPM-1).

Benzyl protected **25** (25.6 mg, 0.024 mol, 1 eq) was dissolved in a mixture of $CHCl_3$:MeOH (1:1, v:v, 2.4 mL) and the solution was purged and bubbled through with a flow of Argon. Then Pd/C (12 mg) was added and the solution was purged and bubbled through with Argon once more. The suspension was bubbled through with Hydrogen and subsequently stirred vigorously under a Hydrogen atmosphere. After 6 hours, the reaction mixture was purged of Hydrogen and filtered over a celite pad. The filtrate was concentrated and suspended in acetone. The suspension was filtered over another celite pad. The product was then eluted by washing the celite pad with a mixture of $CHCl_3$/MeOH/water 9.5:9.5:1 and the filtrate was concentrated in vacuo. Compound **1** was obtained after size exclusion chromatography (LH-20, DCM/MeOH, 1/1, v/v) as a white solid (8.0 mg, 0.024 mmol, 47%). $[\alpha]_D^{20}$= + 6.0 °(c = 0.003, DCM). ^{1}H NMR (500 MHz, $CDCl_3$/MeOD/D_2O 95:95:10) δ: 4.26 – 4.10 (m, 2H, H-1, H-2), 3.92 – 3.78 (m, 2H, OCH_2-lipid), 3.77 – 3.65 (m, 2H, H-6), 3.65 – 3.56 (m, 1H, H-4), 3.45 – 3.38 (m, 1H, H-3), 1.88 – 1.70 (m, 2H, H-7), 1.70 – 1.57 (m, 2H, CH_2-lipid), 1.57 – 1.48 (m, 1H, H-5), 1.48 – 1.16 (m, 36H, CH_2-lipid, CH-lipid), 1.16 – 1.01 (m, 9H, CH_2-lipid, CH-lipid), 0.97 – 0.78 (m, 18H, CH_3-lipid). ^{13}C-APT NMR (126 MHz, $CDCl_3$/MeOD/D_2O 95:95:10) δ: 74.0 (C-3), 73.3 (d, J = 5.76 Hz, C-1), 72.0 (d, J =

3.12 Hz, C-2), 70.4 (C-4), 65.6 (d, J = 5.52 Hz, OCH_2-lipid), 63.1 (C-6), 40.1 (C-5), 36.9 (CH_2-lipid), 36.8 (CH_2-lipid), 36.8 (CH_2-lipid), 36.7 (CH_2-lipid), 36.4 (CH_2-lipid), 32.6 (CH_2-lipid), 32.3 (CH-lipid), 32.2 (CH-lipid), 32.2 (CH-lipid), 32.1 (CH-lipid), 31.4 (CH_2-lipid), 29.4 (CH_2-lipid), 29.0 (CH_2-lipid), 28.8 (CH_2-lipid), 27.9 (CH_2-lipid), 27.8 (C8), 27.6 (C-7), 26.5 (CH_2-lipid), 23.9 (CH_2-lipid), 23.9 (CH_2-lipid), 23.8 (CH_2-lipid), 22.1 (CH_2-lipid), 19.1 (CH_3-lipid), 19.0 (CH_3-lipid), 18.9 (CH_3-lipid), 18.7 (CH_3-lipid), 13.2 (CH_3-lipid). ^{31}P-Hdec NMR (202 MHz, $CDCl_3$/MeOD/D_2O 95:95:10) δ: 1.28. HRMS $[M+H]^+$: 707.5574 found, 707.5541 calculated.

Mannose-1-C-phosphonate mycoketide (MPM-2)

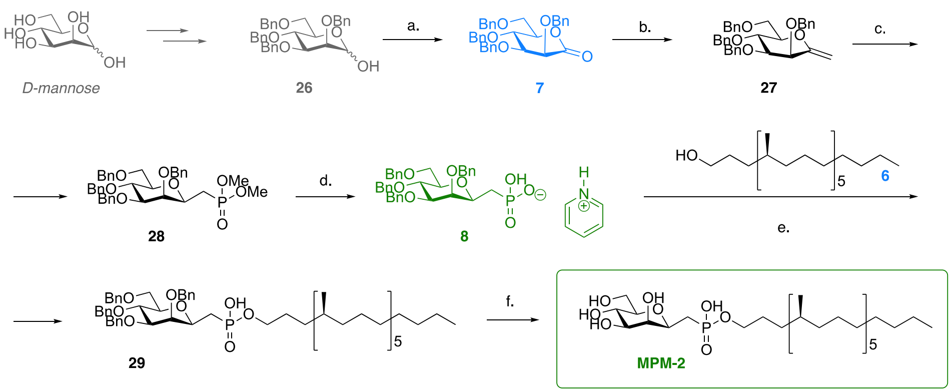

Synthetic scheme for the generation of C-mannoside (MPM-2). a) DMSO, Ac_2O, y: 77%, b) Cp_2TiMe_2, toluene, 60°C, y: 79%, c) $(MeO)_2P(=O)H$, DPAP, neat, hν = 375 nm, y: 59%, d) TMSBr, pyridine, CH_3CN, y: 85%, e) compound **6**, *i*Pr_3PhSO_2Cl, pyridine, 50°C, y: 50%, f) Pd/C, H_2, THF/H_2O (1:1, v/v), y: 80%.

1-one-2,3,4,6-tetra-O-benzyl-D-mannopyranoside (7).

2,3,4,6-tetra-O-benzyl-D-mannopyranoside **26** (23.34 g, 43 mmol, 1 eq) was dissolved in dry DMSO (430 ml) and heated to 30°C. Then Ac_2O (81 ml, 860 mmol, 20 eq) was added and the reaction mixture was stirred overnight at a constant temperature of 30°C. The reaction mixture was diluted with water and Et_2O and transferred to a separatory funnel. The water layer was extracted (4x) with Et_2O and the combined organic phases dried over $MgSO_4$, filtered and concentrated in vacuo. Compound **7** was obtained after crystallization from Et_2O/pentane as a white crystal (17.85 g, 33 mmol, 77%). NMR analysis confirmed purity of the product, whose ^{1}H NMR and ^{13}C NMR spectra were in agreement with published literature. [3]

2,3,4,6-tetra-O-benzyl-1-deoxy-D-manno-hept-1-enitol (27).

Lactone **7** (2.6 g, 4.8 mmol, 1 eq) was co-evaporated (2x) with toluene and dissolved in toluene (24 ml). After protecting the reaction vessel from light, a 5% solution of dimethyltitanocene (44 ml, 9.4 mmol, 1.95 eq) in THF/toluene was added, the reaction

mixture was heated to 60°C and stirred overnight. Since the reaction was not complete, an additional portion of dimethyltitanocene (20 ml, 4.6 mmol, 0.96 eq) was added and the reaction mixture stirred for an additional day. At this point the volatiles were removed in vacuo. Compound **27** was obtained after silicagel chromatography (Pentane/Et_2O 24:1→6:1; DCM loading of crude) as a colorless oil (2.03 g, 3.8 mmol, 79%). NMR analysis confirmed purity of the product, whose ^1H NMR and ^{13}C NMR spectra were in agreement with published literature. [4]

Dimethyl 2,3,4,6-tetra-O-benzyl-β-D-manno-heptulopyranose-1- phosphonate (28).

Focused UV irradiation: A mixture of compound **27** (139 mg, 0.25 mmol, 1 eq), 2,2-dimethoxy-2-phenylacetophenone (27 mg, 0.13 mmol, 0.5 eq) and dimethyl phosphite (2.3 ml, 25 mmol, 100 eq) was stirred and irradiated for 3 hours and 30 minutes using UV light (375 nm). Volatiles were removed in vacuo. Compound **31** was obtained after silicagel chromatography (DCM/acetone 15:1→8:2; DCM/acetone loading of crude) as a colorless oil (385 mg, 0.59 mmol, 59%).

Unfocused sunlight:

A mixture of compound **27** (47 mg, 0.08 mmol, 1 eq), 2,2-dimethoxy-2-phenylacetophenone (10 mg, 0.04 mmol, 0.5 eq) and dimethyl phosphite (0.7 ml, 8 mmol, 100 eq) was stirred for 1 day at RT in the absence of additional UV irradiation. Volatiles were removed in vacuo. Compound **28** was obtained after silicagel chromatography (DCM/acetone 15:1→8:2; DCM/acetone loading of crude) as a colorless oil (22 mg, 0.034 mmol, 43%). ^1H NMR (300 MHz, $CDCl_3$) δ: 7.39 – 7.15 (m, 20H, H-arom), 5.07 (d, J = 11.5 Hz, 1H, CH_2-Bn), 4.87 (d, J = 10.8 Hz, 1H, CH_2-Bn), 4.84 – 4.71 (m, 2H, CH_2-Bn), 4.67 (d, J = 11.5 Hz, 1H, CH_2-Bn), 4.62 – 4.45 (m, 4H, CH_2-Bn), 3.95 – 3.84 (m, 2H, H-4, H-2), 3.84 – 3.71 (m, 2H, H-1, H-3), 3.71 – 3.56 (m, 8H, CH_3-O, H-6), 3.50 (ddd, J = 9.8, 5.1, 2.4 Hz, 1H, H-5), 2.21 (ddd, J = 18.1, 15.4, 6.9 Hz, 1H, CH_2-P), 1.98 (ddd, J = 18.3, 15.4, 6.1 Hz, 1H, CH_2-P). ^{13}C-APT NMR (101 MHz, $CDCl_3$) δ: 138.7 (C-arom), 138.4 (C-arom), 128.6 (C-arom), 128.5 (C-arom), 128.4 (C-arom), 128.4 (C-arom), 128.3 (C-arom), 128.2 (C-arom), 128.0 (C-arom), 127.9 (C-arom), 127.8 (C-arom), 127.7 (C-arom), 127.7 (C-arom), 85.0 (C-3), 79.6 (C-5), 75.94 (d, J = 8.3 Hz, C-2), 75.3 (CH_2-Bn), 75.0 (C-4), 74.7 (CH_2-Bn), 73.5 (CH_2-Bn), 73.4 (C-1), 72.8 (CH_2-Bn), 69.6 (C-6), 52.57 (d, J = 74.0 Hz, CH_3-O), 27.76 (d, J = 140.8 Hz, CH_2-P). ^{31}P-Hdec NMR (121 MHz, $CDCl_3$) δ: 30.7. HRMS $[M+H]^+$: 647.2794 found, 647.2768 calculated.

Pyridinium 2,3,4,6-tetra-O-benzyl-β-D-manno-heptulopyranose-1-phosphonate (8).

Compound **28** (385 mg, 0.59 mmol, 1 eq) was co-evaporated (3x) with toluene, dissolved in dry CH_3CN (39 ml) and cooled to 0°C. A glass stopper was used to seal the reaction vessel and a glass covered stirring rod was used to stir the reaction. Pyridine (0.55 ml, 6.8 mmol, 11.5 eq) and TMSBr (1.56 ml, 11.8 mmol, 20 eq) were added dropwise via syringe. The reaction was heated up to RT and stirred for 2 hours. Volatiles were removed in vacuo, with water bath temperature of 15°C under fume hood. Ice cold milli-q water and acetonitrile were added and after stirring the mixture for 20 minutes, the volatiles were removed in vacuo. Compound **8** was obtained as a white solid (349 mg, 0.5 mmol, 85%) without any further purification. ^1H NMR (400 MHz, MeOD/$CDCl_3$ 3:1) δ: 8.81 – 8.62 (m, 6H, H-pyr), 8.48 – 8.36 (m, 3H, H-pyr), 7.97 – 7.81 (m, 7H, H-pyr), 7.29 – 7.06 (m, 20H, H-

arom), 4.91 (d, J = 10.6 Hz, 1H, CH_2-Bn), 4.76 (d, J = 11.3 Hz, 2H, CH_2-Bn), 4.61 (d, J = 11.1 Hz, 2H, CH_2-Bn), 4.48 (d, J = 10.9 Hz, 1H, CH_2-Bn), 4.35 – 4.26 (m, 2H, CH_2-Bn), 4.11 (d, J = 2.7 Hz, 1H, H-2), 3.90 – 3.77 (m, 2H, H-4, H-1), 3.68 (dd, J = 9.5, 2.8 Hz, 1H, H-3), 3.60 (dd (2X), J = 10.8, 3.1 Hz, 2H, H-6), 3.37 (ddd, J = 9.7, 4.3, 2.0 Hz, 1H, H-5), 2.09 (ddd (2X), J = 15.1, 11.0, 7.5 Hz, 2H, CH_2-P). ^{13}C-APT NMR (101 MHz, MeOD) δ: 148.1 (C-arom), 143.3 (C-arom), 140.3 (C-arom), 139.9 (C-arom), 139.9 (C-arom), 139.4 (C-arom), 130.8 (C-arom), 129.5 (C-arom), 129.5 (C-arom), 129.5 (C-arom), 129.4 (C-arom), 129.4 (C-arom), 129.4 (C-arom), 129.4 (C-arom), 129.3 (C-arom), 129.3 (C-arom), 129.2 (C-arom), 129.0 (C-arom), 128.9 (C-arom), 128.8 (C-arom), 128.8 (C-arom), 128.8 (C-arom), 128.8 (C-arom), 86.0 (C-3), 80.5 (C-5), 77.70 (d, J = 7.3 Hz, C-2), 76.3 (CH_2-Bn), 76.1 (CH_2-Bn), 75.9 (C-4), 75.5 (C-1), 74.5 (CH_2-Bn), 73.3 (CH_2-Bn), 70.6 (C-6), 31.00 (d, J = 137.6 Hz, CH_2-P). 31P-Hdec NMR (202 MHz, MeOD) δ: 26.5. HRMS $[M+H]^+$: 619.2466 found, 619.2455 calculated.

1-phosphoryl-(4S,8S,12S,16S,20S)-4,8,12,16,20-pentamethylheptacosyl-2,3,4,6-tetra-O-benzyl-β-D-manno-heptulopyranose (29).

Phosphonate **8** (100 mg, 0.1 mmol, 2 eq) and lipid **6** (20 mg, 0.04 mmol, 1 eq) were co-evaporated (2x) in toluene and dissolved in dry pyridine (1.4 ml). Tri-iso-propylphenylsulfonyl chloride (45 mg, 0.15 mmol, 3 eq) was added and the reaction mixture was stirred at 50°C overnight. The reaction was quenched with milli-q water and stirred for 2 hours, then EtOAc was added and the reaction mixture was transferred to a separatory funnel. The water layer was extracted (1x) with EtOAc and (2x) with DCM, dried over Na_2SO_4 and concentrated in vacuo. Compound **29** was obtained after silicagel chromatography (DCM/MeOH 20:1→1:1; DCM/MeOH loading of crude; ultrapure silica) and size exclusion (LH-20, DCM/MeOH, 1/1, v/v) as a colorless oil (20 mg, 0.02 mmol, 50%). The pyridinium salt was converted to the sodium salt by dissolving the product in MeOH and treating it with Amberlite Na^+. ^{1}H NMR (600 MHz, $CDCl_3$/MeOD 9:1) δ: 7.38 – 7.21 (m, 18H, H-arom), 7.10 – 6.97 (m, 2H, H-arom), 4.98 (d, J = 11.1 Hz, 1H, CH_2-Bn), 4.83 – 4.72 (m, 2H, CH_2-Bn), 4.70 (d, J = 11.6 Hz, 1H, CH_2-Bn), 4.63 (d, J = 11.1 Hz, 1H, CH_2-Bn), 4.60 – 4.47 (m, 2H, CH_2-Bn), 4.36 (d, J = 10.8 Hz, 1H, CH_2-Bn), 3.85 – 3.77 (m, 2H, H-1, H-2), 3.77 – 3.67 (m, 3H, CH_2-O, H-6a), 3.66 – 3.55 (m, 2H, H-3, H-4), 3.55 – 3.45 (m, 2H, H-6b, H-5), 2.09 – 1.94 (m, 2H, CH_2-P), 1.65 – 1.47 (m, 3H, CH_2-lipid, CH-lipid), 1.37 – 1.17 (m, 33H, CH_2-lipid, CH-lipid), 1.10 – 1.01 (m, 9H, CH_2-lipid, CH-lipid), 0.88 (t, J = 7.0 Hz, 3H, CH_3-lipid), 0.86 – 0.76 (m, 15H, CH_3-lipid). ^{13}C-APT NMR (151 MHz, $CDCl_3$/MeOD 9:1) δ: 138.2 (C-arom), 138.1 (C-arom), 138.0 (C-arom), 137.9 (C-arom), 137.8 (C-arom), 137.0 (C-arom), 128.6 (C-arom), 128.5 (C-arom), 128.5 (C-arom), 128.4 (C-arom), 128.4 (C-arom), 128.4 (C-arom), 128.3 (C-arom), 128.3 (C-arom), 128.2 (C-arom), 128.1 (C-arom), 128.0 (C-arom), 128.0 (C-arom), 127.9 (C-arom), 127.9 (C-arom), 127.8 (C-arom), 127.8 (C-arom), 127.8 (C-arom), 127.7 (C-arom), 127.7 (C-arom), 84.5 (C-3), 78.1 (C-5), 77.3 (C-2), 75.2 (CH_2-Bn), 75.2 (CH_2-Bn), 75.0 (C-1), 74.8 (C-4), 72.9 (CH_2-Bn), 72.5 (CH_2-Bn), 68.6 (C-6), 64.8 (CH_2-O), 37.5 (CH_2-lipid), 37.5 (CH_2-lipid), 37.5 (CH_2-lipid), 37.5 (CH_2-lipid), 37.4 (CH_2-lipid), 37.4 (CH_2-lipid), 37.4 (CH_2-lipid), 37.1 (CH_2-lipid), 33.2 (CH_2-lipid), 32.9 (CH-lipid), 32.9 (CH-lipid), 32.8 (CH-lipid), 32.8 (CH-lipid), 32.8 (CH_3-lipid), 32.0 (CH_2-lipid), 30.0 (CH_2-P), 29.7 (CH_2-lipid), 29.5 (CH_2-lipid), 29.5 (CH_2-lipid), 29.4 (CH_2-P), 29.1 (CH_2-lipid), 28.9 (CH_2-lipid), 28.8 (CH_2-lipid), 28.7 (CH_2-lipid), 28.6 (CH_2-lipid), 28.2 (CH_2-lipid),

27.1 (CH_2-lipid), 24.6 (CH_2-lipid), 24.5 (CH_2-lipid), 24.5 (CH_2-lipid), 24.5 (CH_2-lipid), 22.7 (CH_2-lipid), 19.8 (CH_3-lipid), 19.8 (CH_3-lipid), 19.7 (CH_3-lipid), 19.5 (CH_3-lipid), 19.5 (CH_3-lipid), 14.1 (CH_3-lipid). ^{31}P-Hdec NMR (162 MHz, $CDCl_3$/MeOD 9:1) δ: 25.6, 24.9. HRMS $[M+H]^+$: 1067.7461 found, 1067.7463 calculated.

1-phosphoryl-(4S,8S,12S,16S,20S)-4,8,12,16,20-pentamethylheptacosyl-β-D-manno-heptulopyranose (MPM-2).

Compound **29** (20 mg, 0.02 mmol, 1 eq) was dissolved in a mixture of THF/H_2O 1:1 and the solution was purged and bubbled through with a flow of Argon. Then Pd/C (10 mg) was added and the solution was purged and bubbled through with Argon once more. The suspension was bubbled through with Hydrogen and subsequently stirred vigorously under a Hydrogen atmosphere. After 16 hours, the reaction mixture was purged of Hydrogen and filtered over a Whatman filter. Compound **2** was obtained after in vacuo removal of volatiles as an amorphous solid (12 mg, 0.016 mmol, 80%). ^{1}H NMR (850 MHz, $CDCl_3$/MeOD/D_2O 95:95:10) δ: 3.91 (d, J = 2.5 Hz, 1H, H-2), 3.88 – 3.78 (m, 4H, H-6a, CH_2-O, H-1), 3.71 (dd, J = 12.0, 5.6 Hz, 1H, H-6b), 3.60 – 3.53 (m, 2H, H-3, H-4), 3.28 (ddt, J = 8.0, 5.6, 2.3 Hz, 1H, H-5), 2.01 – 1.96 (m, 1H, CH_2-P), 1.95 – 1.92 (m, 1H, CH_2-P), 1.69 – 1.56 (m, 4H, CH_2-lipid), 1.43 – 1.15 (m, 43H, CH_2-lipid, CH-lipid), 1.12 – 1.04 (m, 8H, CH_2-lipid), 0.92 – 0.81 (m, 18H, CH_3-lipid). ^{13}C-APT NMR (214 MHz, $CDCl_3$/MeOD/D_2O 95:95:10) δ: 79.8 (C-5), 74.4 (C-1), 74.3 (C-3), 71.19 (d, J = 7.5 Hz, C-2), 66.7 (C-4), 64.32 (d, J = 5.6 Hz, CH_2-O), 60.9 (C-6), 37.0 (CH_2-lipid), 36.9 (CH_2-lipid), 36.9 (CH_2-lipid), 36.9 (CH_2-lipid), 36.9 (CH_2-lipid), 36.8 (CH_2-lipid), 36.8 (CH_2- lipid), 36.5 (CH_2-lipid), 32.7 (CH_2-lipid), 32.3 (CH-lipid), 32.3 (CH-lipid), 32.3 (CH-lipid), 32.2 (CH-lipid), 32.2 (CH-lipid), 31.4 (CH_2-lipid), 29.4 (CH_2-lipid), 29.2 (CH_2-lipid), 29.2 (CH_2-lipid), 28.9 (CH_2-lipid), 28.8 (CH_2-lipid), 28.2 (CH_2- lipid), 28.1 (CH_2-lipid), 26.5 (CH_2-lipid), 24.0 (CH_2-lipid), 24.0 (CH_2-lipid), 23.9 (CH_2-lipid), 23.9 (CH_2-lipid), 23.1 (CH_2-lipid), 22.2 (CH_2-lipid), 19.2 (CH_3-lipid), 19.1 (CH_3-lipid), 19.1 (CH_3-lipid), 19.0 (CH_3-lipid), 18.8 (CH_3-lipid), 13.3 (CH_3- lipid). ^{31}P-Hdec NMR (202 MHz, $CDCl_3$/MeOD/D_2O 95:95:10) δ: 23.0, 22.5. HRMS $[M+H]^+$: 707.5596 found, 707.5585 calculated.

Mannose-1-*C*-difluorophosphonate mycoketide (MPM-3)

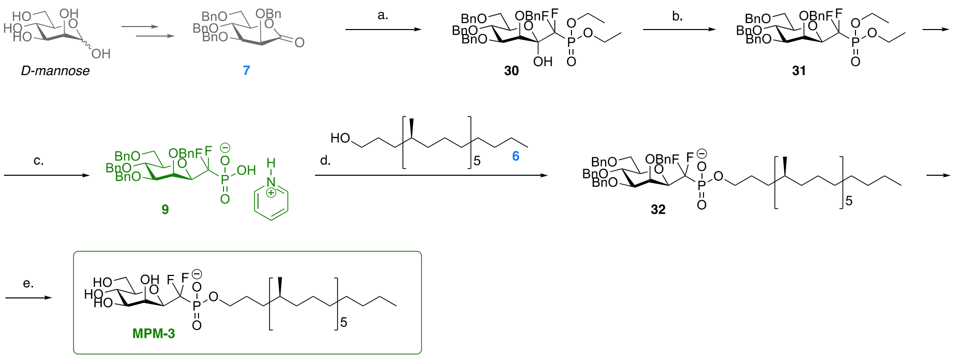

Synthetic scheme for the generation of difluoro-*C*-mannoside (MPM-3). a) LDA, $(EtO)_2P(=O)CHF_2$, THF, y: 98%, b) i. $MeO_2CC(=O)Cl$, DCM, ii. AIBN, Bu_3SnH, toluene, y: 30%, c) TMSBr, pyridine, CH_3CN, y: quant., d) compound **6**, $i$$Pr_3PhSO_2Cl$, toluene:DMF:pyridine (1.25:1:0.5, v/v/v), 50°C, y: 64%, 6) Pd/C, H_2, THF:H_2O (2:1, v/v), y: 82%.

Diethyl 1-hydroxy-2,3,4,6-tetra-O-benzyl-β-D-manno-heptulopyranose-1-(difluoro)phosphonate (30).

A solution of DIPA (1.6 ml, 11 mmol, 2 eq) in THF (30 ml) was cooled to -78°C. After the addition of n-BuLi (6.9 ml, 11 mmol, 2 eq) dropwise via syringe, the reaction mixture was rapidly brought to 0°C for 10 minutes and then cooled again to -78°C. A solution of diethyl (difluoromethyl)phosphonate (1.4 ml, 9.3 mmol, 1.7 eq) in THF (10 ml) was cooled to -78°C and added dropwise via cannula to the first solution. After 1 hour and 30 minutes, a solution of lactone **7** (2.96 g, 5.5 mmol, 1 eq) in THF (10 ml) was cooled to -78°C and added dropwise via cannula to the reaction mixture. After 10 minutes from the addition of the last drop of lactone the reaction was stirred for additional 10 minutes with a saturated solution of NH_4Cl(aq) and finally diluted with EtOAc and transferred to a separatory funnel. The water layer was extracted (3x) with EtOAc and the combined organic layers were washed (1x) with water and (1x) with brine, dried over Na_2SO_4 and concentrated in vacuo. Compound **30** was obtained after silicagel chromatography (Pentane/EtOAc 10:1→2:1; DCM loading of crude) as a colorless syrup (3.95 g, 5.4 mmol, 98%). ^{1}H NMR (500 MHz, $CDCl_3$) δ: 7.40 – 7.17 (m, 20H, H-arom), 6.00 (s, 1H, -OH), 4.87 (d, J = 10.9 Hz, 1H, CH_2-Bn), 4.82 (d, J = 11.0 Hz, 1H, CH_2-Bn), 4.78 – 4.66 (m, 3H, CH_2-Bn), 4.60 – 4.51 (m, 2H, CH_2-Bn), 4.42 (d, J = 11.8 Hz, 1H, CH_2-Bn), 4.34 – 4.23 (m, 4H, CH_2-O), 4.20 (bs, 1H, H-2), 4.17 – 4.09 (m, 2H, H-3, H-5), 3.99 (t, J = 9.7 Hz, 1H, H-4), 3.79 (dd, J = 11.2, 5.9 Hz, 1H, H-6a), 3.69 (dd, J = 11.4, 1.7 Hz, 1H, H-6b), 1.38 (t, J = 7.1 Hz, 3H, CH_3), 1.17 (t, J = 7.1 Hz, 3H, CH_3). ^{13}C NMR (126 MHz, $CDCl_3$) δ: 138.6 (C-arom), 138.5 (C-arom), 138.4 (C-arom), 138.3 (C-arom), 128.5 (C-arom), 128.4 (C-arom), 128.4 (C-arom), 128.2 (C-arom), 128.1 (C-arom), 128.1 (C-arom), 127.8 (C-arom), 127.7 (C-arom), 127.6 (C-arom), 127.6 (C-arom), 127.6 (C-arom), 127.4 (C-arom), 116.56 (ddd, J = 286.9, 265.2, 194.0 Hz, CF2), 96.61 (ddd, J = 30.6, 19.1, 11.1 Hz, C-1), 81.08 (d, J = 2.6 Hz, C-3), 75.2 (C-2), 75.2 (CH_2-Bn), 74.7 (CH_2-Bn), 74.6 (C-4), 73.1 (CH_2-Bn), 73.1 (C-5), 72.4 (CH_2-Bn), 69.5

(C-6), 65.71 (dd, J = 50.5, 6.3 Hz, CH_2-O), 16.35 (dd, J = 15.2, 5.9 Hz, CH_3). ^{19}F NMR (471 MHz, $CDCl_3$) δ: -118.38 (dd, J = 304.5, 96.1 Hz), -119.83 (dd, J = 304.5, 100.1 Hz). ^{31}P NMR (202 MHz, $CDCl_3$) δ: 8.14 (dd, J = 100.0, 96.0 Hz). HRMS $[M+Na]^+$: 749.2659 found, 749.2662 calculated.

Diethyl 2,3,4,6-tetra-O-benzyl-β-D-manno-heptulopyranose-1-(difluoro)phosphonate (31).

Compound **30** (3.6 g, 5 mmol, 1 eq) was co-evaporated with toluene and dissolved in a dry mixture of DCM/pyridine 5:1 (25 ml) before being cooled to 0°C. Methyl oxalyl chloride (0.92 ml, 10 mmol, 2 eq) was dissolved in DCM (2.5 ml) and slowly added to the first solution via syringe before an extra portion of pyridine was added (2.5 ml). The reaction mixture was warmed up to RT and stirred for 10 minutes before quenching it with EtOH over 10 minutes. Then a saturated solution of $NaHCO_3$ and DCM were used to dilute the reaction mixture before it was transferred to a separatory funnel. The organic layer was concentrated in vacuo and the reaction intermediate was dissolved in dry toluene (250 ml) without further purification. This solution was purged and bubbled through with Argon before the addition of Bu_3SnH and AIBN. The reaction mixture was heated up to reflux and stirred overnight. After removal of volatiles in vacuo, the crude was purified via silicagel chromatography (Pentane/EtOAc 9:1→3:1), followed by treatment with a 1 M KF(aq) solution and silicagel/KCO_3 9:1 chromatography (Pentane/EtOAc 3:2). Compound **31** was obtained after silicagel chromatography as a colorless syrup (1.05 g, 1.48 mmol, 30% over two steps). ^{1}H NMR (500 MHz, $CDCl_3$) δ: 7.44 – 7.39 (m, 2H, H-arom), 7.35 – 7.23 (m, 16H, H-arom), 7.20 – 7.17 (m, 2H, H-arom), 4.91 – 4.84 (m, 2H, CH_2-Bn), 4.79 (d, J = 11.2 Hz, 1H, CH_2-Bn), 4.70 (d, J = 11.7 Hz, 1H, CH_2-Bn), 4.64 (d, J = 11.8 Hz, 1H, CH_2-Bn), 4.60 – 4.54 (m, 2H, CH_2-Bn), 4.47 (d, J = 11.8 Hz, 1H, CH_2-Bn), 4.30 – 4.18 (m, 5H, CH_2-O, H-2), 3.98 (t, J = 9.6 Hz, 1H, H-4), 3.91 (dt, J = 22.3, 3.4 Hz, 1H, H-1), 3.78 – 3.69 (m, 2H, H-6), 3.63 – 3.56 (m, 2H, H-3, H-5), 1.36 – 1.31 (m, 3H, CH_3), 1.21 – 1.16 (m, 3H, CH_3). ^{13}C NMR (126 MHz, $CDCl_3$) δ: 138.6 (C-arom), 138.3 (C-arom), 138.2 (C-arom), 138.1 (C-arom), 128.5 (C-arom), 128.5 (C-arom), 128.4 (C-arom), 128.3 (C-arom), 128.2 (C-arom), 128.2 (C-arom), 128.1 (C-arom), 127.9 (C-arom), 127.8 (C-arom), 127.8 (C-arom), 127.7 (C-arom), 127.6 (C-arom), 127.5 (C-arom), 117.40 (ddd, J = 280.4, 257.0, 209.8 Hz, CF2), 84.05 (d, J = 1.9 Hz, C-3), 80.6 (C-5), 75.74 (ddd, J = 31.4, 18.8, 13.6 Hz, C-1), 75.3 (CH_2-Bn), 74.7 (C-4), 74.4 (CH_2-Bn), 73.3 (CH_2-Bn), 72.34 (dd, J = 6.1, 2.1 Hz, C-2), 72.2 (CH_2-Bn), 69.5 (C-6), 64.96 (dd, J = 30.3, 6.5 Hz, CH_2-O), 16.43 (dd, J = 9.5, 5.7 Hz, CH_3). ^{31}P NMR (162 MHz, $CDCl_3$) δ: 6.73 (dd, J = 101.2, 99.2 Hz). ^{19}F NMR (471 MHz, $CDCl_3$) δ: -116.13 (dd, J = 312.3, 100.9 Hz), -124.45 (ddd, J = 312.4, 99.0, 22.3 Hz). HRMS $[M+Na]^+$: 733.2716 found, 733.2712 calculated.

Pyridinium 2,3,4,6-tetra-O-benzyl-β-D-manno-heptulopyranose-1-(difluoro)phosphonate (9).

Compound **31** (71 mg, 0.1 mmol, 1 eq) was co-evaporated (3x) with toluene, dissolved in dry CH_3CN (6.7 ml) and cooled to 0°C. A glass stopper was used to seal the reaction vessel and a glass-covered stirring rod was used to stir the reaction. Pyridine (0.1 ml, 1.2 mmol, 12 eq) and TMSBr (0.26 ml, 2 mmol, 20 eq) were added dropwise via syringe. The reaction was heated up to RT and stirred overnight. Volatiles were removed in vacuo, with water

bath temperature of 20°C under the fume hood. Ice cold milli-q water and acetonitrile were added and after stirring the mixture for 2 hours and 30 minutes, the volatiles were removed in vacuo. Compound **9** was obtained as a white solid (70 mg, 0.1 mmol, quant.) without any further purification. ^{1}H NMR (500 MHz, MeOD) δ: 8.95 – 8.85 (m, 12H, H-pyr), 8.69 – 8.62 (m, 6H, H-pyr), 8.11 – 8.03 (m, 12H, H-pyr), 7.50 – 7.45 (m, 2H, H-arom), 7.39 – 7.24 (m, 15H, H-arom), 7.22 – 7.17 (m, 3H, H-arom), 7.11 – 7.06 (m, 2H, H-arom), 4.97 – 4.91 (m, 3H, CH_2-Bn), 4.79 – 4.74 (m, 2H, CH_2-Bn), 4.66 (d, J = 11.0 Hz, 2H, H-2, CH_2-Bn), 4.29 – 4.11 (m, 4H, CH_2-Bn, H-4, H-1), 3.89 (dd, J = 9.4, 2.7 Hz, 1H, H-3), 3.81 (dd, J = 11.0, 1.9 Hz, 1H, H-6a), 3.74 (dd, J = 11.0, 3.5 Hz, 1H, H-6b), 3.64 (ddd, J = 9.9, 3.6, 1.9 Hz, 1H, H-5). ^{13}C NMR (126 MHz, MeOD) δ: 148.5 (C-arom), 147.0 (C-arom), 143.0 (C-arom), 140.2 (C-arom), 140.0 (C-arom), 140.0 (C-arom), 139.2 (C-arom), 138.9 (C-arom), 129.9 (C-arom), 129.8 (C-arom), 129.8 (C-arom), 129.8 (C-arom), 129.7 (C-arom), 129.7 (C-arom), 129.7 (C-arom), 129.7 (C-arom), 129.6 (C-arom), 129.6 (C-arom), 129.5 (C-arom), 129.5 (C-arom), 129.4 (C-arom), 129.3 (C-arom), 129.2 (C-arom), 129.2 (C-arom), 129.1 (C-arom), 129.0 (C-arom), 129.0 (C-arom), 129.0 (C-arom), 128.9 (C-arom), 128.9 (C-arom), 85.7 (C-3), 81.4 (C-5), 78.12 (ddd, J = 27.8, 11.8, 4.7 Hz, C-1), 76.2 (CH_2-Bn), 76.1 (CH_2-Bn), 75.9 (C-4), 74.7 (C-2), 74.3 (CH_2-Bn), 73.4 (CH_2-Bn), 70.1 (C-6). ^{19}F NMR (471 MHz, MeOD) δ: -118.58 (ddd, J = 302.1, 88.8, 8.1 Hz), -123.20 (ddd, J = 302.2, 94.6, 19.9 Hz). ^{31}P NMR (202 MHz, MeOD) δ: 3.36 (dd, J = 94.7, 89.0 Hz). HRMS $[M+Na]^+$: 655.2268 found, 655.2267 calculated.

1-(difluoro)phosphoryl-(4S,8S,12S,16S,20S)-4,8,12,16,20-pentamethylheptacosyl-2,3,4,6-tetra-O-benzyl-β-D-manno- heptulopyranose (32).

Compound **9** (7 mg, 0.01 mmol, 1 eq) and lipid 10 (16 mg, 0.018 mmol, 3 eq) were co-evaporated (2x) in toluene and dissolved in a mixture of dry DMF/pyridine 2:1 (0.24 ml) and dry toluene (0.20 ml) respectively. This solution was heated up to 60°C before the addition of tri-iso-propylphenylsulfonyl chloride (5.6 mg, 0.018 mmol, 1.8 eq). The reaction mixture was then stirred overnight at this temperature and then concentrated. Compound **32** was obtained after silicagel chromatography ($CHCl_3$/MeOH 40:1→9:1; $CHCl_3$/MeOH 40:1 loading of crude; ultrapure silica, neutralized with 1% Et_3N) as a colorless oil (7 mg, 0.0064 mmol, 64%). ^{1}H NMR (500 MHz, MeOD) δ: 7.64 – 6.96 (m, 20H, H-arom), 4.87 (d, J = 10.9 Hz, 2H, CH_2-Bn), 4.81 (d, J = 11.6 Hz, 1H, CH_2-Bn), 4.74 (d, J = 10.7 Hz, 1H, CH_2-Bn), 4.71 (d, J = 11.6 Hz, 1H, CH_2-Bn), 4.59 (d, J = 11.4 Hz, 2H, CH_2-Bn), 4.50 (d, J = 11.7 Hz, 1H, CH_2-Bn), 4.41 (bs, 1H, H-2), 4.06 – 3.93 (m, 2H, H-1, H-4), 3.93 – 3.81 (m, 6H, -OCH_3), 3.80 – 3.70 (m, 3H, H-3, H-6), 3.66 – 3.56 (m, 1H, H-5). ^{13}C NMR (126 MHz, MeOD) δ: 140.2 (C-arom), 140.0 (C-arom), 140.0 (C-arom), 139.8 (C-arom), 129.7 (C-arom), 129.6 (C-arom), 129.6 (C-arom), 129.5 (C-arom), 129.5 (C-arom), 129.4 (C-arom), 129.4 (C-arom), 129.2 (C-arom), 129.0 (C-arom), 129.0 (C-arom), 128.9 (C-arom), 128.8 (C-arom), 85.4 (d, J = 2.1 Hz, H-3), 81.8 (C-5), 77.6 – 77.0 (m, C-1), 76.4 (CH_2-Bn), 76.1 (C-4), 76.0 (CH_2-Bn), 74.5 (CH_2-Bn), 74.22 – 74.12 (m, C-2), 73.5 (CH_2-Bn), 70.7 (C-6), 56.3 (d, J = 6.4 Hz, -OCH_3), 56.0 (d, J = 6.7 Hz, -OCH_3). ^{31}P NMR (202 MHz, MeOD) δ: 9.85 (pt, J = 102.0 Hz). ^{19}F NMR (471 MHz, MeOD) δ: -114.97 (ddd, J = 313.6, 101.5, 4.3 Hz), -123.23 (ddd, J = 313.5, 102.7, 21.8 Hz). HRMS $[M+H]^+$: 1103.7279 found, 1103.7275 calculated.

1-(difluoro)phosphoryl-(4S,8S,12S,16S,20S)-4,8,12,16,20-pentamethylheptacosyl-β-D-manno-heptulopyranose (MPM-3).

Compound **32** (12 mg, 0.011 mmol, 1 eq) was dissolved in a mixture of THF/H_2O 2:1 (5 ml) and the solution was purged and bubbled through with a flow of Argon. Then Pd/C (10 mg) was added and the solution was purged and bubbled through with Argon once more. The suspension was bubbled through with Hydrogen and subsequently stirred vigorously under a Hydrogen atmosphere. After 16 hours, the reaction mixture was purged of Hydrogen and filtered over celite. Compound **3** was obtained after in vacuo removal of volatiles as an amorphous solid (7 mg, 0.009 mmol, 82%). ^{1}H NMR (500 MHz, MeOD) δ: 4.28 (d, J = 3.1 Hz, 1H, H-2), 4.10 – 3.94 (m, 2H, OCH_2), 3.89 (pd, J = 12.3 Hz, 1H, H-6a), 3.87 – 3.61 (m, 4H, H-1, H-6b, H-4), 3.57 – 3.49 (m, 1H, H-3), 3.31 – 3.26 (m, 1H, H-5), 3.18 (q, J = 7.3 Hz, 4H, CH_2-triethylammonium), 1.77 – 1.54 (m, 4H, CH_2-lipid), 1.45 – 1.17 (m, 54H, CH_2-lipid, CH-lipid, CH_3-triethylamonium), 1.12 – 1.02 (m, 9H, CH_2-lipid, CH-lipid), 0.90 – 0.84 (m, 18H, CH_3-lipid). ^{13}C NMR (126 MHz, MeOD) δ: 81.0 (C-5), 79.0 (C-1), 74.2 (C-3), 67.3 (C-4), 67.0 (OCH_2), 66.4 (C-4), 60.8 (C-6), 46.2 (CH_2-triethylammonium), 36.9 (CH_2-lipid), 36.8 (CH_2-lipid), 36.7 (CH_2-lipid), 36.4 (CH_2-lipid), 32.4 (CH_2-lipid), 32.3 (CH-lipid), 32.2 (CH-lipid), 32.2 (CH-lipid), 31.4 (CH_2-lipid), 29.4 (CH_2-lipid), 29.1 (CH_2-lipid), 28.8 (CH_2-lipid), 26.5 (CH_2-lipid), 23.9 (CH_2-lipid), 23.9 (CH_2-lipid), 23.8 (CH_2-lipid), 22.1 (CH_2-lipid), 19.1 (CH_3-lipid), 19.1 (CH_3-lipid), 19.1 (CH_3-lipid), 19.0 (CH_3-lipid), 18.7 (CH_3-lipid), 13.3 (CH_3-lipid), 7.9 (CH_3-triethylammonium). ^{31}P NMR (202 MHz, MeOD) δ: 2.84 (t, J = 85.4 Hz). ^{19}F NMR (471 MHz, MeOD) δ: -115.15 (dd, J = 305.6, 86.7 Hz), -117.29 (dd, J = 305.5, 84.5 Hz). HRMS $[M+H]^+$: 683.2577 found, 683.2580 calculated.

References

[1] Shoji Akai, Rika Tanaka, Hidekazu Hoshi, and Kenichi Sato. Selective deprotection method of n-phenylcarbamoyl group. The Journal of organic chemistry, 78(17) :8802–8808, 2013.

[2] Robert V Stick and Keith A Stubbs. The synthesis of a new class of potential inhibitors for glycoside hydrolases. Journal of carbohydrate chemistry, 24(4-6): 529–547, 2005.

[3] Julie Gratien, Marie-Pierre Heck, and Charles Mioskowski. C-2 epimerization of aldonolactones promoted by magnesium iodide: a new way towards non-enzymatic epimerization. Carbohydrate research, 343(1) :18–30, 2008.

[4] Daniel Waschke, Julian Thimm, and Joachim Thiem. Highly efficient synthesis of ketoheptoses. Organic letters, 13(14):3628–3631, 2011.

Supplementary Figure 1: NOESY NMR Spectra

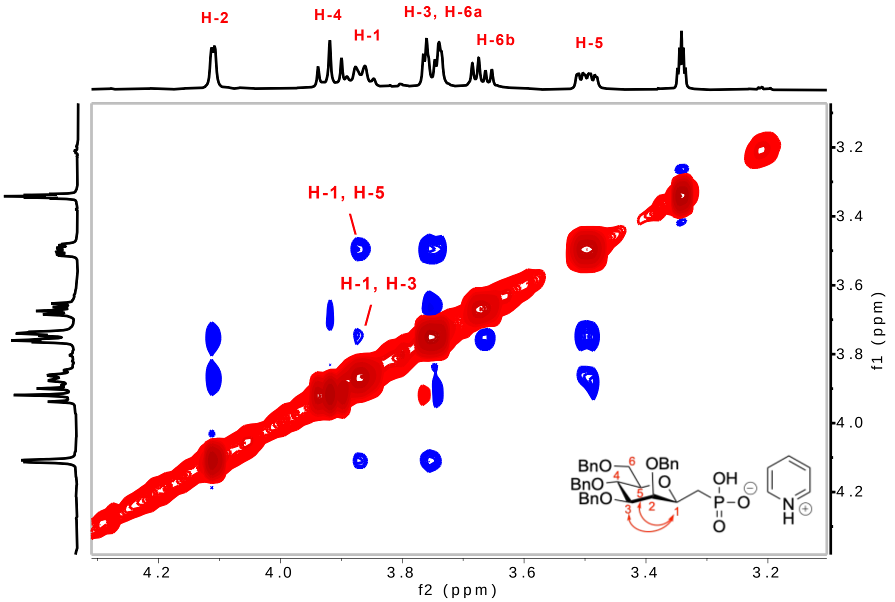

A NOESY spectrum of compound 8. The key NOE interaction can be found between H-1 and H-5, H-1 and H-3.

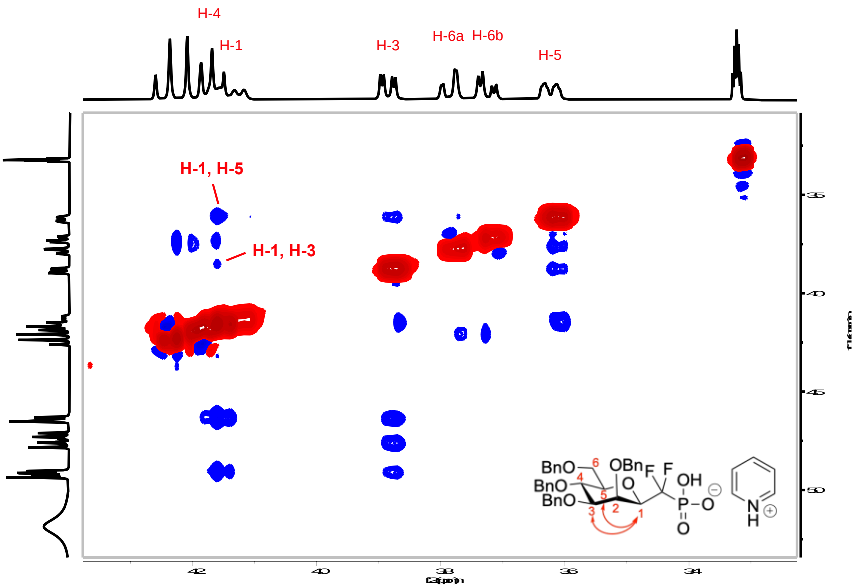

B NOESY spectrum of compound 9. The key NOE interaction can be found between H-1 and H-5, and H-1 and H-3.

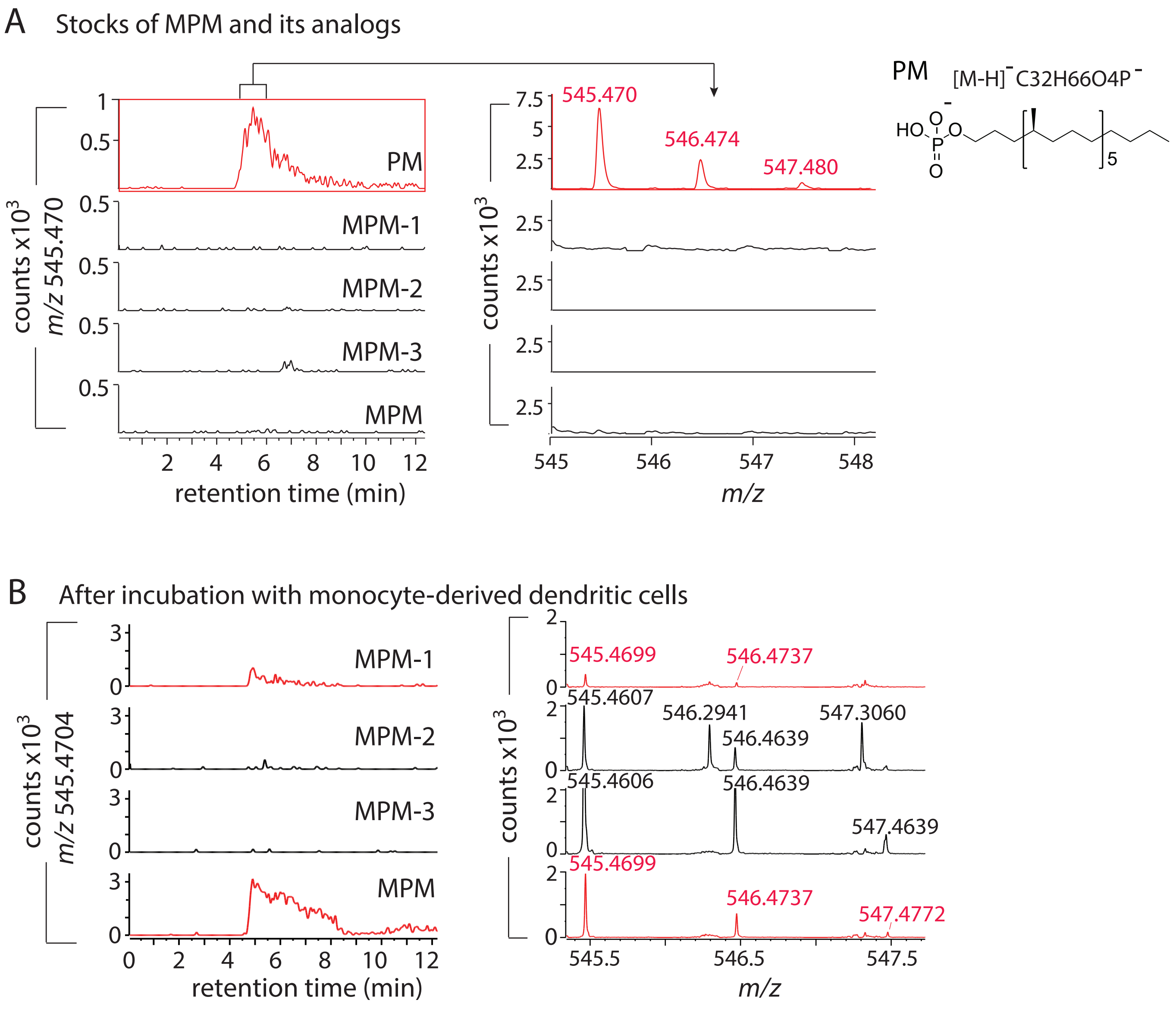

Supplementary Figure 2 Hydrolysis of MPM and analogs. A: MPM and analogs were analyzed for the presence of PM by HPLC-MS. Left panel: extracted ion chromatogram. Right: mass spectrum at the elution time of PM. B: Monocyte-derived dendritic cells were exposed to MPM or its analogs for 24 h. Lipids were extracted from cell pellets and analyzed for the presence of PM by HPLC-MS. Red: compounds that match *m/z* values that, within experimental error, were consistent with PM. Black: m/z values that did not match PM.

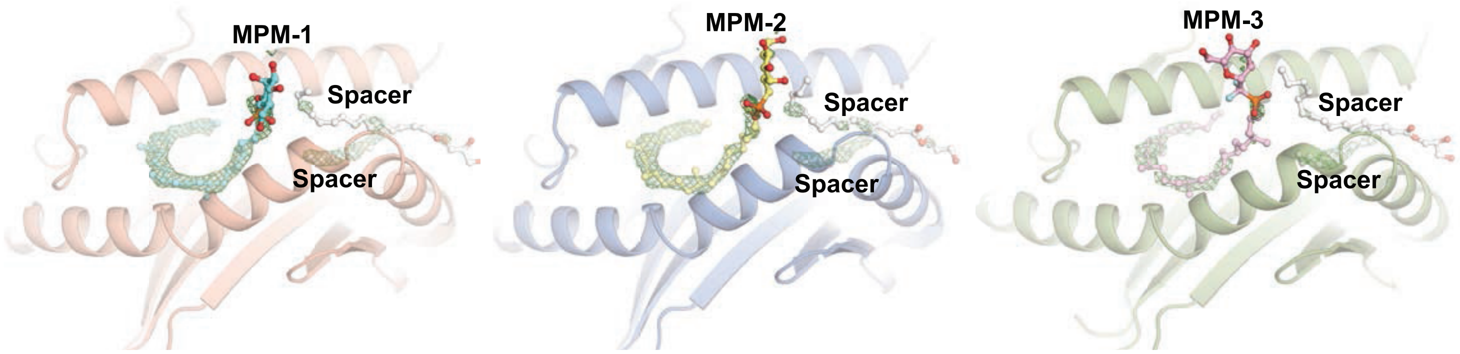

Supplementary Figure 3. Unbiased electron density maps (green mesh) of MPM analogues in CD1c binding cleft are contoured at 2.2 σ. Cartoon representation of CD1c is coloured as salmon, blue, and green for structure with MPM-1, MPM-2, and MPM-3, respectively. MPM analog models are depicted as sticks with balls coloured with aquamarine, yellow, and pink for MPM-1, MPM-2, and MPM-3, respectively. Spacer lipids are in white.

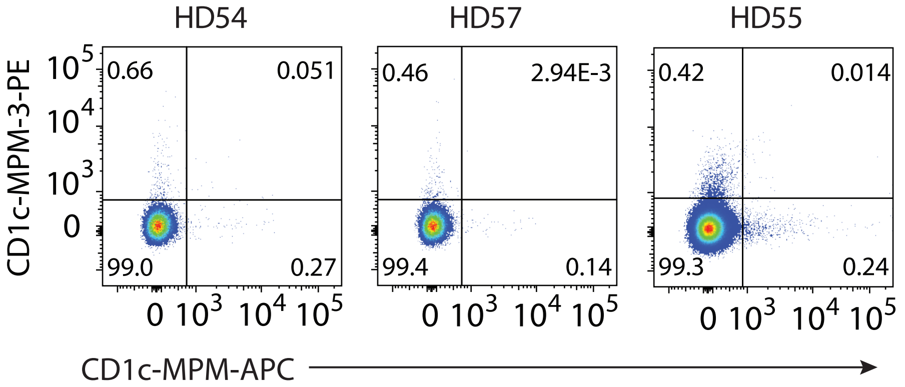

Supplementary Figure 4. Lack of detection of a CD1c-MPM-3 or CD1c-MPM tetramer-positive population in three healthy donors after in vitro immunization with MPM-3.

Supplementary Table 1. Data collection and refinement statistics

	CD1c MPM-1	CD1c MPM-2	CD1c MPM-3
PDB ID	7MX4	7MXF	7MXH
**Data collection**			
Resolution (Å)	48.56 - 1.73 (1.79 - 1.73)	43.67 - 2.0 (2.07 - 2.0)	44.16 - 2.11 (2.19 - 2.11)
Space group	$P\ 2_1\ 2_1\ 2_1$	$P\ 2_1\ 2_1\ 2_1$	$P\ 2_1\ 2_1\ 2_1$
Cell dimensions			
a, b, c (Å)	55.42, 71.74, 100.76	55.22, 71.36, 100.03	55.57, 72.73, 98.92
α, β, γ (°)	90, 90, 90	90, 90, 90	90, 90, 90
Total reflections	85360 (8416)	54834 (5424)	47466 (4686)
Unique reflections	42680 (4208)	27417 (2711)	23737 (2342)
Multiplicity	2.0 (2.0)	2.0 (2.0)	2.0 (2.0)
Completeness (%)	99.8 (99.3)	100 (100)	99.9 (99.9)
Mean $I/\sigma(I)$	11.6 (1.7)	12.3 (2)	8.3 (1.7)
Wilson B-factor	27.3	33.2	33.6
R_{meas} (%)	2.8 (46.7)	3.9 (46.5)	6.4 (61.9)
R_{pim} (%)	2.0 (33.1)	2.8 (32.9)	4.5 (43.8)
$CC_{1/2}$ (%)	100 (79.6)	99.9 (77.9)	99.5 (63.9)
**Refinement**			
R_{work} (%)	20.7	20.5	20.6
R_{free} (%)	23.9	24.2	25.2
Number of non-hydrogen atoms	3413	3306	3318
Macromolecules	3067	3052	3084
Ligands	124	117	113
Water	222	137	121
Protein residues	383	381	385
R.m.s deviations			
Bond length (Å)	0.007	0.008	0.008
Bond angle (°)	1.06	1.05	1.12
Ramachandran plot			
Favoured (%)	95.8	96.8	96.3
Allowed (%)	3.4	2.7	3.4
Outliers (%)	0.8	0.5	0.3
Average B-factor	33.7	39.1	39.1
Macromolecules	32.7	38.6	38.3
Ligands	47.3	52.3	54.9
Water	36.0	38.8	39.1

*Values in parentheses are for highest-resolution shell.
